# Analysis of phylogenetic relationships in *Macadamia* shows evidence of extensive reticulate evolution

**DOI:** 10.3389/fpls.2024.1394244

**Published:** 2024-10-15

**Authors:** Sachini Lakmini Manatunga, Agnelo Furtado, Bruce Topp, Mobashwer Alam, Patrick J. Mason, Ardashir Kharabian-Masouleh, Robert J. Henry

**Affiliations:** ^1^ Queensland Alliance for Agriculture & Food Innovation (QAAFI), University of Queensland, St Lucia, QLD, Australia; ^2^ ARC Centre of Excellence for Plant Success in Nature and Agriculture, The University of Queensland, St Luci, QLD, Australia; ^3^ Queensland Alliance for Agriculture & Food Innovation (QAAFI), Maroochy Research Facility, The University of Queensland, Nambour, QLD, Australia

**Keywords:** *Macadamia*, phylogenetic, wild, chloroplast, nuclear genes, chloroplast capture

## Abstract

The genus *Macadamia* in the Proteaceae family includes four species native to Australia. Two of the four species, *M. integrifolia* and *M. tetraphylla*, have recently been utilized to generate domesticated macadamia varieties, grown for their edible nuts. To explore diversity in macadamia genetic resources, a total of 166 wild genotypes, representing all four species, were sequenced. The four species were clearly distinguished as four separate clades in a phylogenetic analysis of the nuclear genome (based upon concatenated nuclear gene CDS and SNPs). The two larger species (*M. integrifolia* and *M. tetraphylla*) formed a clade, that had diverged from a clade including the smaller species (*M. ternifolia* and *M. jansenii*). The greatest diversity in nuclear and chloroplast genomes was found in the more widely distributed *M. integrifolia* while the rare *M. jansenii* showed little diversity. The chloroplast phylogeny revealed a much more complex evolutionary history. Multiple chloroplast capture events have resulted in chloroplast genome clades, including genotypes from different species. This suggests extensive reticulate evolution in *Macadamia* despite the emergence of the four distinct species that are supported by the analysis of their nuclear genomes. The chloroplast genomes showed strong associations with geographical distribution reflecting limited maternal gene movement in these species that have large seeds. The nuclear genomes showed lesser geographical differences, probably reflecting the longer distance pollen movement. This improved understanding of the distribution of diversity in *Macadamia* will aid in the conservation of these rare species now found in highly fragmented rainforest remnants.

## 1 Introduction

Phylogenetics, the study of evolutionary relationships among organisms, has become a powerful tool in a variety of biological disciplines (Liu et al., 2022). Over the last few decades, substantial effort has been made to understand the phylogenetic associations among angiosperms through the application of DNA sequence data (Jansen et al., 2006). Next-generation sequencing (NGS) has brought a transformation in sequence analysis by making it more affordable and increasing access to complete chloroplast and nuclear genome data (Zhou et al., 2021). There has been an emphasis by breeders to exploit wild germplasm, but a significant portion remains underutilized (Zhang and Batley, 2020). The lack of genomic data on wild germplasm is one barrier to its effective use in plant breeding efforts, and as a result, the integration of genes from wild germplasm into cultivated varieties has been limited. Recent advancements in genomics and bioinformatics have provided opportunities to unlock the hidden potential within wild germplasm (Tanksley, 1997) by extending this technology to less-studied plant species. This has opened new avenues to incorporate materials from wild germplasm (Zhang and Batley, 2020).

Macadamia is an evergreen perennial rainforest tree of the family Proteaceae and is indigenous to Australia (Hardner et al., 2009; Walton, 2011; O’Connor et al., 2019). The genus *Macadamia* is the only angiosperm that has been domesticated as a large-scale commercial food crop in Australia (Aradhya et al., 1998). In accordance with the present classification of Proteaceae (Mast et al., 2008), the genus *Macadamia* has been classified into four species, namely, *M. integrifolia* (Maiden & Betche), *M. tetraphylla* (L.A.S. Johnson), *M. ternifolia* (F. Muell), and *M. jansenii* (C.L. Gross & P.H. Weston), using molecular and morphological data, while many species previously classified as *Macadamia* have been transferred to other genera. Among the four species, *M. integrifolia* has the widest natural distribution, extending from southeast Queensland to the New South Wales border. Two overlapping distributions lead to natural hybridization between *M. integrifolia* and *M. tetraphylla* and between *M. integrifolia* and *M. ternifolia* (O’Connor et al., 2019; Topp et al., 2019). *M. tetraphylla* is mostly distributed in New South Wales, while *M. ternifolia* is distributed north of Brisbane, extending from the Samford Valley to Gympie (Topp et al., 2019). *M. jansenii* is the most geographically isolated species and is found only in Bulburin National Park north of Bundaberg, 180 km from the closest *M. integrifolia* population (Topp et al., 2019; Mai et al., 2020). The genus *Macadamia* displays diversity in several morphological characteristics. These include the number of leaves per whorl, mature leaf size and shape, color of new leaves, presence of petiole, leaf margin serration, and differences in floral and fruit morphology (Peace, 2005). These characteristics are used for differentiating *Macadamia* species. However, some of these characteristics, such as leaf serration, can overlap across species and can be observed only at a certain stage (i.e., juvenile or adult) of the life cycle of some species. On the other hand, traits like nut and leaf size can vary within species depending on the environment and may not always be useful in distinguishing between the species (Peace, 2005; Hardner et al., 2009). Genomic information on the representative accessions of these four species can be instrumental in understanding the diversity and species distribution of *Macadamia.*

Although genomic investigation in macadamia commenced a decade ago, only a few studies have been conducted to date. Several types of polymorphic molecular markers have been used to assess the genetic diversity in *Macadamia* (Peace, 2005; Mast et al., 2008; Ahmad Termizi et al., 2016; Alam et al., 2018; O’Connor et al., 2019; Mai et al., 2020). However, few studies have been employed to characterize the genetic makeup of wild germplasm (Mai et al., 2020). In 2005, Peace et al. studied a large number of wild germplasm accessions using low-throughput RAF (randomly amplified DNA fingerprinting) and RAMiFi (randomly amplified microsatellite fingerprinting) markers (Peace, 2005). Another study by Mast et al. (2008) investigated the relationships between the four *Macadamia* species and their closely related wild relatives. They examined chloroplast DNA regions, such as matK, atpB, and ndhF and nuclear DNA genomic regions, such as waxy loci 1 and 2 and PHYA. By analyzing these markers, their aim was to gain insights into the complex relationships within the *Macadamia* genus and its wild relatives (Mast et al., 2008). However, these markers gave low genome coverage and provided poor marker density (Alam et al., 2018; Nock et al., 2020). Ahmad Termizi et al. (2016) analyzed individuals from wild *M. integrifolia* population using 516 single-nucleotide polymorphisms (SNPs) and reported the unique chlorotypes for each of the 12 samples (Ahmad Termizi et al., 2016). Furthermore, a recent study (Mai et al., 2020) examined the genetic relationships among 302 wild germplasm accessions using 2,872 SNPs and 8,415 *in silico* DArT markers and identified the species status of 94 unknown wild accessions. Although these studies examined the phylogenetic relationships among wild macadamia accessions, no previous study has resolved the phylogeny of the four *Macadamia* species.

In *Macadamia*, as in other plants, uniparentally inherited chloroplast DNA has been used to infer the phylogenetic patterns. However, many studies have documented the occurrence of reticulate evolution of chloroplast in other plant species (Nge et al., 2021). The phenomenon of reticulate evolution may result in the replacement of chloroplast genomes of one species with another (Kawabe et al., 2018) due to hybridization events. In many plants, reticulate evolution has caused a discordance between the molecular data derived from the chloroplast and the nuclear genome (Nge et al., 2021), resulting in conflicting topologies for phylogenetic trees (Rieseberg and Soltis, 1991). Therefore, reticulate evolution can have an impact on phylogenetic analyses that rely only on the chloroplast genomes or their genes (Kawabe et al., 2018). However, studies to date have not explored phylogenetic relationships in *Macadamia* based on both nuclear and complete chloroplast genomes.

Here, we focused on uncovering the diversity and relationships in wild *Macadamia* populations by using both chloroplast genomes and nuclear gene coding sequences (CDS). This is the first whole-genome sequencing data report for a large *Macadamia* population. To support improved conservation and utilization of the wild genetic resources, we sequenced whole chloroplast and nuclear genomes to better understand diversity within and relationships between species and populations of *Macadamia* in Australia.

## 2 Materials and methods

### 2.1 Plant materials, DNA extraction, and sequencing

A total of 166 wild macadamia accessions representing all four species [*M. integrifolia* (*n* = 49), *M. tetraphylla* (*n* = 56), *M. ternifolia* (*n* = 23), and *M. jansenii* (*n* = 23)] and one related rainforest species from the Proteaceae, *Lasjia whelanii*, were selected for sequencing. Within the macadamia populations, 161 wild accessions, which were collected previously from multiple locations across the natural distribution of the four species, were grown and maintained at Nambour arboretum and *ex situ* germplasm centers at Nambour and Tiaro in Queensland and Alstonville in NSW (Hardner et al., 2004) and five from a private collection at Limpinwood, NSW (**Supplementary Table 1**) in Australia. Fully expanded young macadamia leaves, of accession within these *ex situ* collection sites, were collected in perforated labeled cellophane bags and immediately placed under dry ice until stored in a −80°C freezer at The University of Queensland, Brisbane, Australia.

Frozen leaves were coarse pulverized under liquid nitrogen using a mortar and pestle and further fine pulverized under cryogenic conditions using a Qiagen tissue lyser (MM400, Retsch, Germany). A modified version of the cetyltrimethylammonium bromide (CTAB) DNA extraction protocol described by Furtado (2014) was used to extract genomic DNA. The quality and quantity of the DNA samples were evaluated using a Nanodrop spectrophotometer (Nanodrop Technologies, Wilmington, DE, USA) by recording the absorbance ratios at 260/280 and 260/230 followed by running a 0.7% agarose gel with SYBR safe staining (Thermo Fisher Scientific). Whole-genome short-read sequencing was undertaken by BGI Hong Kong. A PCR-free library was generated and sequencing at 150-bp paired-end reads was undertaken on the DNBSEQ-G400 sequencing platform from MGI (MGI Tech Co., Ltd, Shenzhen, China) at an expected data yield/sample of at least 25× genome size (800 Mb genome)

### 2.2 Chloroplast genome assembly and annotation

All sequence data were analyzed in CLC Genomics Workbench 23.0.05 (CLC-GWB, CLC-Bio, QIAGEN, Denmark, http://www.clcbio.com) using the short-read pipeline. Quality control (QC) was performed for all short-read data. Reads were trimmed using a quality score limit of 0.01 with default parameters (more than 98% of the resulted trimmed reads had a Phred score >25). A subset of quality trimmed short reads (2–13 GB) was used for chloroplast genome assembly. All chloroplast genomes were assembled using the GetOrganelle toolkit (Jin et al., 2020) exploiting SPAdes v.3.15.3, Bowtie2 v.2.4.5, and Blast v.2.11.0 as dependencies. The correct configuration of the chloroplast genome was selected with respect to the *M. integrifolia* (sequence: NC_025288.1) (Nock et al., 2014) available at the National Center for Biotechnology Information (NCBI) (http://www.ncbi.nlm.nih.gov/) using clone manager software (Sci Ed, USA).

The chloroplast genomes were annotated using the GeSeq online tool (https://chlorobox.mpimp-golm.mpg.de/geseq.html) with *M. integrifolia* (sequence: NC_025288.1) as the genome (Tillich et al., 2017). Chloroplast genomes were annotated with the following settings: Annotation options: Annotate plastid inverted repeat (IR), Annotated plastid trans spliced rps12, Annotation support: Support annotation by Chloe, Annotation revision: Keep best annotation only, BLAT search–protein search identity: 25, rRNA, tRNA, and DNA search identity: 85, HMMER profile: CDS+rRNA, ARAGORN v1.2.38- Genetic code: Bacterial/plant chloroplast, Max intron length: 3,000, tRNAscan-se v2.0.7- sequence source: organellar tRNAs, MPI-MP reference set: chloroplast land plants (CDS + rRNA) and Chloe v0.1.0- annotate- CDS + tRNA + rRNA. All genomes were imported to Geneious 2023.2.1 software (Biomatters Ltd, USA) to determine the number of genes, CDS, transfer RNAs (tRNAs), and ribosomal RNAs (rRNAs) in each sample.

### 2.3 Concatenated nuclear gene CDS sequences

Previously published annotated sequences of the nuclear genome of *M. integrifolia* (Nock et al., 2020) were selected as reference sequences to generate accession-specific consensus CDS of nuclear genes. CDS of *M. integrifolia* (GCF 013358625.1) were downloaded and imported to CLC-GWB to generate a local Blast database. The CDS of 106 *Arabidopsis thaliana* single-copy genes identified by Li et al. (2017) was subjected to tblastn against the *M. integrifolia* CDS database. We selected 56 tblastn hits with a single *M. integrifolia* CDS matching an *A. thaliana* CDS as these hits represented single-copy genes in the *M. integrifolia* genome. From these selected single hits, corresponding *M. integrifolia* CDS sequences were extracted and used as a reference to extract consensus sequences from each of the macadamia accessions and from *L. whelanii*. BLAST analysis using the 56 extracted *M. integrifolia* CDS and the CDS sequences of *L. whelanii* as a database resulted in the selection of 53 *L. whelanii* CDS as single hits that represented single-copy genes in *L. whelanii*. Corresponding (same as selected) 53 CDS sequences from *M. integrifolia* and from *L. whelanii* were selected for further analysis. The 53 CDS from *M. integrifolia* (**Supplementary Table 2**) were used as reference sequences in the read mapping approach to generate corresponding CDS consensus sequences for each of the macadamia accessions. Essentially, short reads trimmed data of each macadamia accession were mapped separately to each of the 53 selected *M. integrifolia* CDS sequences to extract consensus sequences. Finally, consensus CDS sequences, were extracted for each macadamia accession, and were concatenated in the same sequential order to obtain the final nuclear gene CDS sequence.

### 2.4 Phylogenetic evaluation

Phylogenetic evaluation of macadamia was conducted by utilizing complete chloroplast genome sequences and single-copy concatenated nuclear gene CDS sequences. For phylogenetic analysis, we selected 138 wild macadamia accessions representing all four species. To maintain precision and clarity within our analysis, we exclude accessions from planted wild germplasm, accessions known to be natural hybrids, admixtures, accessions of unknown origin, and unidentified macadamia accessions (**Supplementary Table 3**). The phylogenetic trees were also generated for four species: *M. integrifolia* (*n* = 44), *M. tetraphylla* (*n* = 49), *M. ternifolia* (*n* = 22), and *M. jansenii* (*n* = 23) separately based on chloroplast genomic data and single-copy nuclear gene sequences. *L. whelanii* was used as an outgroup. All selected sequences were aligned along with the outgroup using MAFFT alignment with default parameters in Geneious 2023.2.1 software (Biomatters Ltd, USA).

#### 2.4.1 Chloroplast phylogenetic analysis

Chloroplast genomes are widely used in plant phylogenetic analysis (Yanfei et al., 2023). Therefore, phylogenetic trees were constructed using complete chloroplast genome sequences to investigate the relationships in genus *Macadamia*. To better determine the relationships within the Macadamia species, we first constructed Maximum Likelihood (ML) trees and Bayesian trees for all four species separately using *L. whelanii* as an outgroup.

Chloroplast phylogenetic reconstructions were performed using PAUP*v 4.0 software (Swofford and Sullivan, 2003) with the maximum likelihood (ML) method and MrBayes v. 3.2 software (Ronquist et al., 2012) in Geneious for Bayesian inference (BI) method. For PAUP* trees, the Akaike Information Criterion (AIC) in the jModel test was performed in Cyberinfrastructure for Phylogenetic Research (CIPRES) Science Gateway (https://www.phylo.org/) to find out the best-fitting nucleotide substitution model (Miller et al., 2010). ML analysis was performed with 1,000 bootstrap replicates. GTR + Gamma was used in BI analysis. The chloroplast ML tree for the *M. integrifolia* and *M. tetraphylla* populations were generated by the TPM1uf+I+G model and chloroplast ML tree for *M. ternifolia* were generated by the TVM+G model. The topological structures of trees were evaluated based on bootstrap support and Bayesian posterior probabilities. The Interactive Tree of Life (iTOL) v.5 tool (Letunic and Bork, 2021) (https://itol.embl.de/about.cgi) was used to visualize the phylogenies.

To further evaluate the phylogenetic relationship between the four species of macadamia, we constructed Bayesian tree methods (GTR + Gamma model) by taking 138 complete macadamia chloroplast genomes using *L. whelanii* as an outgroup.

#### 2.4.2 Nuclear gene phylogenetic analysis

##### 2.4.2.1 Concatenation based phylogeny

Concatenated 53 nuclear gene CDS sequences of *Macadamia* species and *L. whelanii* were used to evaluate the phylogenetic relationships in the genus *Macadamia*. The nuclear gene phylogenetic trees were generated by using Randomized Axelerated Maximum Likelihood (RAxML) version 8 (Stamatakis, 2014) with the ML method and the BI method in MrBayes v. 3.2 software (Ronquist et al., 2012) in Geneious 2023.2.1 software (Biomatters Ltd, USA). ML trees were analyzed using the GTR + GAMMA nucleotide model with 1,000 bootstrap replicates. The BI trees were analyzed using the GTR + GAMMA model. The phylogenetic trees were visualized with the iTOL v.5 tool (Letunic and Bork, 2021). The topological structure of trees produced by RAxML and MrBayes software was compared to identify discrepancies between them.

##### 2.4.2.2 Single Nucleotide Polymorphisms (SNPs) based phylogeny

The CDS from 53 genes from *M. integrifolia* (**Supplementary Table 2**) were analysed by BLASTed against CDS extracted for *M. integrifolia* (GWHESFF00000000), *M. tetraphylla* (GWHESFG00000000), *M. ternifolia* (GWHESEN00000000) and *M. jansenii* (GWHESFI00000000) (Sharma et al., 2023) from Genome warehouse (https://ngdc.cncb.ac.cn/gwh/) in CLC Genomics Workbench 23.0.05 (CLC-GWB, CLC-Bio, QIAGEN, Denmark, http://www.clcbio.com). The BLAST hits were manually checked. We selected 45 BLAST hits with a single hit for each species (**Supplementary Table 4**). Then, short reads were trimmed using a 0.01 quality threshold. All the samples were independently mapped to the respective reference genomes of the four species. The mapping setting was as follows: Masking mode (No), Match score (1), Mismatch score (2), Insertion cost (3), Deletion cost (3), Length fraction (1.0), Similarity fraction (0.9) and Global alignment (No). To identify variants, present in selected 45 CDS, the read mappings were then subjected to a Fixed ploidy variant detection tool in CLC Genomics Workbench 23.0.05 (CLC-GWB, CLC-Bio, QIAGEN, Denmark, http://www.clcbio.com). Fixed ploidy variant detection was conducted at the settings as follows; minimum coverage (10), minimum count (3) and minimum frequency (%) (25%). Then, Heterozygous SNP variants were manually filtered for a frequency range of 25 – 75% for the two alleles with respect to the reference genomes. Homozygous SNP variants were manually filtered for 100% frequency. Variants for outgroup *L. whelanii* were identified with respect to the *M. integrifolia* (GWHESFF00000000) reference genome. Individual heterozygous and 100% homozygous variant files were combined to produce a comma separated value format (CSV) tables for each of the 45 CDS in all samples. The CSV files with a final set of SNPs were used to reconstruct CDS sequences for each sample using a Python script (https://github.com/Aeyohan/aght). Reconstructed CDS sequences were then aligned with the outgroup using MAFFT alignment with default parameters in Geneious 2023.2.1 software (Biomatters Ltd, USA). Individual gene trees were generated using RAxML version 8 (Stamatakis, 2014) with the ML method in Geneious 2023.2.1 software (Biomatters Ltd, USA). ML trees were analysed using the GTR +GAMMA nucleotide model with 1000 bootstrap replicates. Then, individual gene trees were used to construct ASTRAL tree (Zhang et al., 2018). The Interactive Tree Of Life (iTOL) v.5 tool (Letunic and Bork, 2021) (https://itol.embl.de/about.cgi) was used to visualize the phylogeny.

### 2.5 Assessment of phylogeography

Geographical maps of origin were created for all four macadamia species based on the chloroplast phylogenetic clade separation. Geographic ranges were mapped using Esri National Geographic in the QGIS Geographic Information System (Version 3.32.1-Lima) (http://qgis.osgeo.org). Geographical coordinates of each accession are listed in **Supplementary Table 3**.

## 3 Results

### 3.1 Characterization of chloroplast genomes

Following paired-end sequencing (150 bp), a total of 140,789,058–260,592,896 reads were obtained for 166 *Macadamia* accessions (**Supplementary Table 5**) with sequence depth of 18.23× and 50×. The sequence depth of trimmed paired-end reads at a quality score limit of 0.01 ranged between 16.47× and 43.98×. Two assembled sequences were obtained from GetOrganelle analysis indicating the presence of two structural haplotypes of the chloroplast genome that occurs in plants related to the orientation of the single-copy region. The correct configuration of the chloroplast genome was selected with respect to *M. integrifolia* (Reference sequence: NC_025288.1). Complete chloroplast genome sizes analyzed in this study are shown in **Supplementary Table 6**. Complete circular chloroplast genomes were obtained for all genotypes, ranging in size from 159,195 to 159,734 bp. The smallest chloroplast genome was identified for three *M. tetraphylla* accessions (Mac_297, Mac_338, and Mac_345) and two wild macadamia trees of uncertain species (Mac_047 and Mac_329) while the largest was observed for two *M. integrifolia* accessions (Mac_029 and Mac_262). Chloroplast genome sizes of *M. integrifolia* ranged from 159,458 to 159,734 bp, those of *M. ternifolia* ranged from 159,463 to 159,508 bp, those of *M. tetraphylla* ranged from 159,195 to 159,598 bp, and *M. jansenii* were 159,524 bp in length except for MacP_16 (159,526 bp). The result of this study also revealed that out of 23 *M. jansenii* accessions, 22 accessions had identical chloroplast genomes.

**Figure 1:**
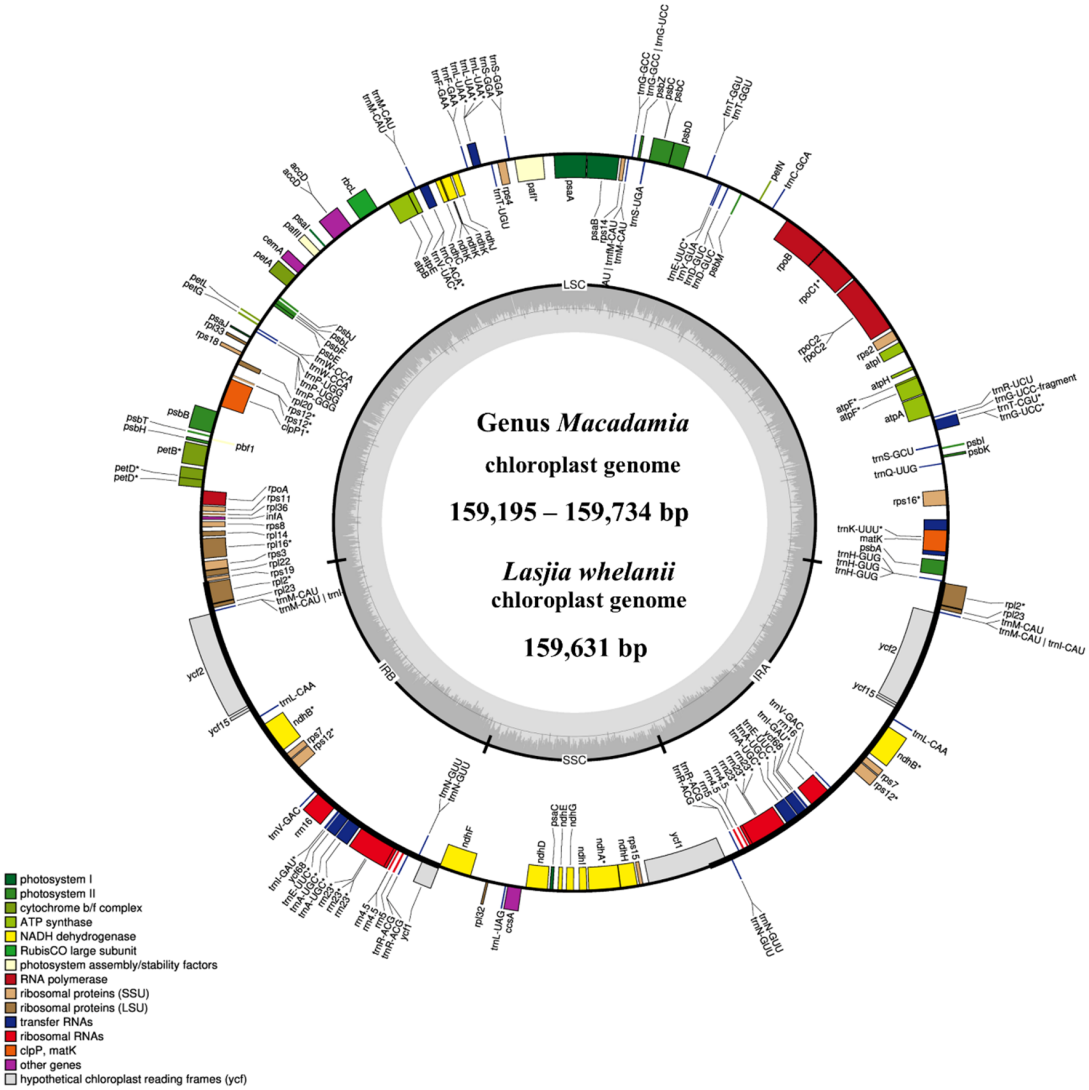
The circular chloroplast genome map of four *Macadamia* species and *Lasjia whelanii*. Genes inside the circle are transcribed in the clockwise direction whereas the genes outside the circle are transcribed in the counterclockwise direction. Genes belonging to different functional groups are colour coded. Gray area in the inner circle indicates the GC content of the chloroplast genome. The four regions of a chloroplast genome are also indicated in the inner circle: the two inverted repeat regions (IRA and IRB) are separated by small (SSC) and large (LSC) single copy regions.

All macadamia chloroplast genomes showed a quadripartite structure of angiosperm, including a large single copy (LSC), a small single copy (SSC), and two identical inverted repeats (IRa and IRb) (**Figure 1**). Gene annotation showed 116 full-length genes, 81 CDS, 4 rRNAs, and 31 tRNAs. Among these 116 genes, 60 genes were involved in protein synthesis and DNA replication (genes responsible for rRNAs, tRNAs, large subunit of ribosome, small subunit of ribosome, and DNA-dependent RNA polymerase), 46 were involved in photosynthesis (genes responsible for subunits of photosystem I, subunits of photosystem II, subunits of ATP synthase, subunits of NADH dehydrogenase, large subunit of rubisco, and subunits of cytochrome complex), 6 were involved in other different functions (genes responsible for inner membrane protein, cytochrome synthesis gene, acetyl-CoA-carboxylase, maturase, ATP-dependent protease, and translational initiation factor), and 4 were involved in unknown function genes (**Supplementary Table 7**).

The *L. whelanii* chloroplast genome possessed the standard quadripartite structure, containing two inverted repeats (18,824 bp), the LSC region (87,911 bp), and the SSC region (26,448 bp). (**Figure 1**). Genome size was recorded as 159,631 bp. The plastome of *L. whelanii* contained no significant difference in relation to genes, protein coding genes, rRNA, and tRNA. Overall, the GC content of the chloroplast genome was recorded as 38%.

### 3.2 Chloroplast phylogeny and geographical analysis of *Macadamia* species

**Figure 2:**
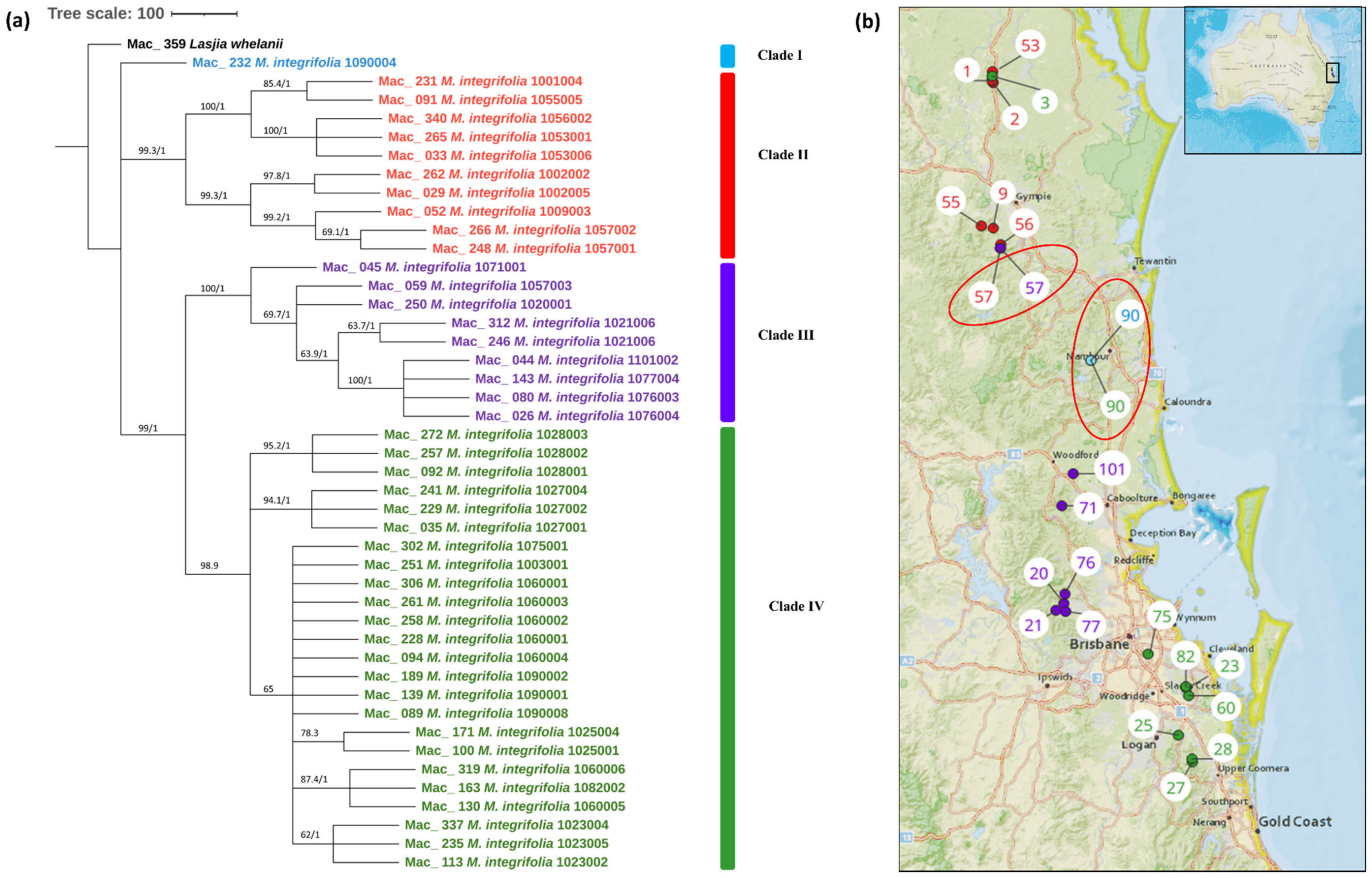
**(A)** Chloroplast phylogeny of *M. integrifolia* using the ML and BI method. **(B)** Phylogeographic analysis of *M. integrifolia*. Numbers above the lines represent ML bootstrap support/Bayesian posterior probabilities. Numbers indicate corresponding population site (**Supplementary Table 3**). Light blue: Clade I, Red: Clade II, Purple: Clade III and Green: Clade IV. Coloured dots on the map indicate the corresponding clade in chloroplast phylogenetic tree. Two red circles highlight population site number which contained accessions from different clades.

The multiple chloroplast genome alignment of 44 *M. integrifolia* accessions together with the outgroup *L. whelanii* was 161,281 bp in length with 99.6% identical sites. The tree topologies of both were similar (**Figure 2A**), and most nodes were supported by high bootstrap support (BS) (>95%) and Bayesian posterior probabilities (PP) (>0.95). However, some internal nodes tended to have low BS, indicating incomplete lineage sorting. The phylogenetic tree construction revealed that Mac_232 (corresponding to population site 90) clusters separately from the rest of the 43 accessions. The remaining accessions were differentiated into two main clades and further differentiated into sub-clades. Clade II contained accessions from the northern distribution of *M. integrifolia*: Mac_231, Mac_262, Mac_029, Mac_265, and Mac_033 from the Gundiah/Mount Bauple region (corresponding to population sites 1, 2, 2, 3, and 3, respectively) (**Figure 2B**) and Mac_052, Mac_091, Mac_340, Mac_248, and Mac_266 from the Gympie region (corresponding to population sites 9, 55, 56, 57, and 57, respectively). Clade III included a total of nine accessions, of which seven were from the Caboolture region: Mac_250, Mac_312, Mac_246, Mac_045, Mac_080, Mac_026, and Mac_143 (corresponding to population sites 20, 21, 21, 71, 76, 76, and 77, respectively) and one from the Nambour region: Mac_044 (corresponding to population site 101). Interestingly, Mac_059 from population site 57 did not cluster with two other accessions (Mac_248 and Mac_266) from population site 57 (Gympie region) in Clade I. Clade IV contained 24 accessions that were collected from the region south of Brisbane except for Mac_251, Mac_089, Mac_139, and Mac_189 (corresponding to population sites 3, 90, 90, and 90, respectively). This observation verified that the accessions having the same geographical origin tend to form distinct clusters among themselves. However, it is noteworthy that certain accessions from these localities exhibit a tendency to associate or cluster with accessions sourced from different geographical areas. The result of this study also revealed that Mac_251, Mac_089, Mac_139, and Mac_189 (corresponding to population sites 3, 90, 90, and 90, respectively) are likely to be planted trees. Mac_312, Mac_246 and Mac_306, Mac_228 are biological replicates and confirmed by clustering together. Additionally, the short branch length of the tree suggested that Mac_232 had less divergence while Mac_026, Mac_044, Mac_080, and Mac_143 from Clade III were more diverged accessions.

**Figure 3:**
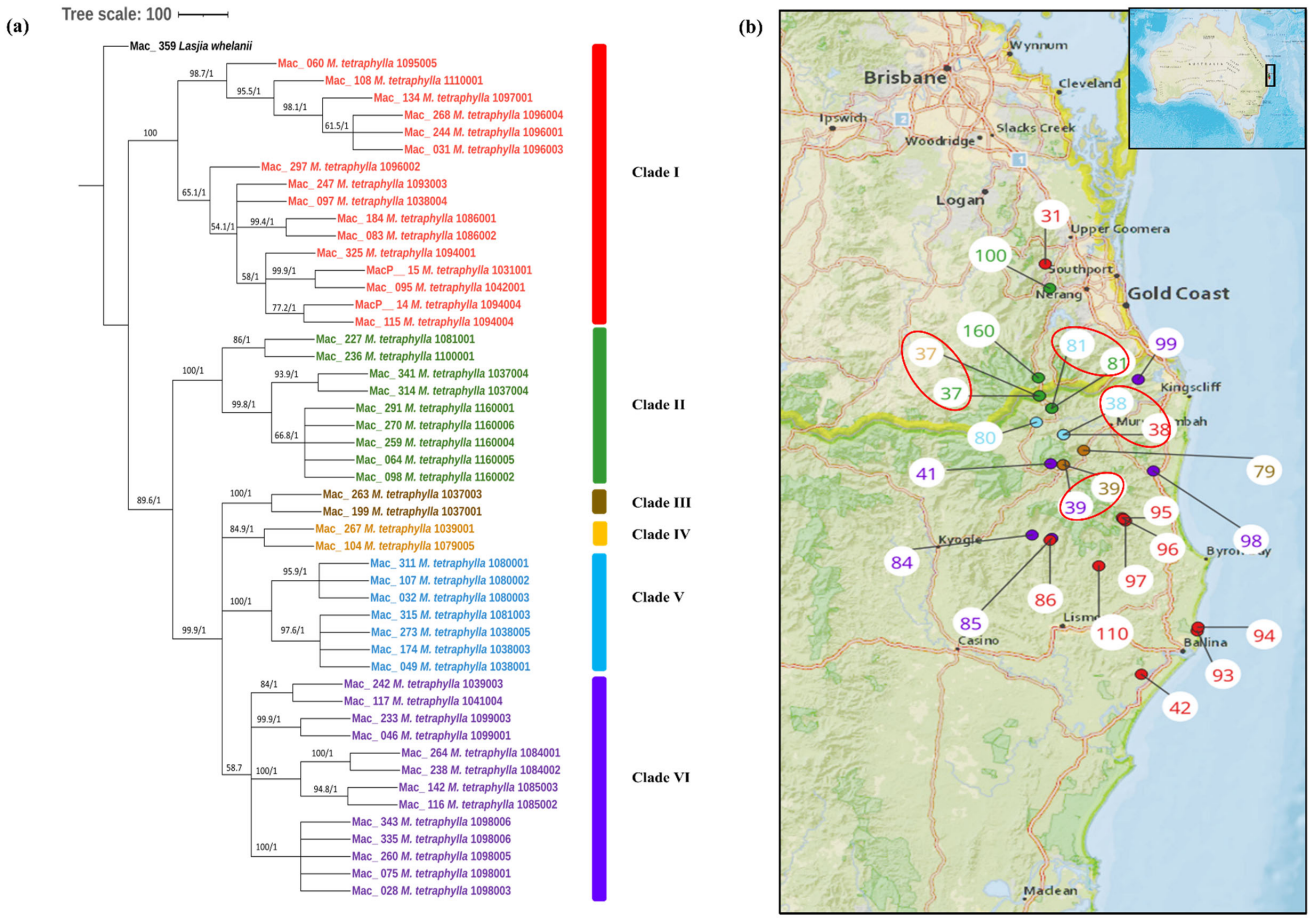
**(A)** Chloroplast phylogeny of *M. tetraphylla* using the ML and BI method. **(B)** Phylogeographic analysis of *M. tetraphylla*. Numbers above the lines represent ML bootstrap support/Bayesian posterior probabilities. Numbers indicate corresponding population site (**Supplementary Table 3**). Red: Clade I, Green: Clade II, Brown: Clade IIII, Orange: Clade IV, Light blue: Clade V and Purple: Clade VI. Coloured dots on the map indicate the corresponding clade in chloroplast phylogenetic tree. Four red circles highlight population site number which contained accessions from different clades.

To study the phylogenetic relationship of *M. tetraphylla*, phylogenetic trees were constructed using 49 complete chloroplast genome sequences. The multiple chloroplast genome alignment of *M. tetraphylla* accessions together with the outgroup *L. whelanii* was 161,554 bp in length with 96% identical sites. ML and BI trees exhibited similar phylogenetic topologies (**Figure 3A**). The resulting phylogenetic tree showed strong statistical support for most internal and external nodes but barring poor BS value for some internal nodes. However, in the Bayesian chloroplast tree, the highest PP value of 1 was observed for all the nodes. The chloroplast tree displayed two major clades. The first major clade (Clade I) consisted of germplasm collected from the southern part: the Lismore region (Mac_060, Mac_108, Mac_134, Mac_268, Mac_244, Mac_031, Mac_297, Mac_184, Mac_083, and Mac_095) and the Ballina region (Mac_247, Mac_325, MacP_14, and Mac_115) except for MacP_15 (corresponding to population site 31) and Mac_097 (corresponding to population site 38) (**Figure 3B**). The second major clade was further divided into sub-clades. Clade II contained accessions from the Murwillumbah region: Mac_341, Mac_314, Mac_227, Mac_270, Mac_098, Mac_064, Mac_291, and Mac_259 (corresponding to population sites 37, 37, 81, 160, 160, 160, 160, and 160, respectively) and the Beenleigh region: Mac_236 (corresponding to population site 100). Two accessions from Clade III from population site 37 clustered separately from the rest of the accessions from the same geographical location. However, as in the *M. integrifolia* chloroplast phylogenetic tree, the majority of *M. tetraphylla* tended to form distinct clusters among themselves based on geographical areas. Results also indicated that Mac_031, Mac_244, and Mac_268 from population site 96 (Clade I) and Mac_264 and Mac_238 from population site 84 (Clade VI) were highly diverged accessions.

**Figure 4:**
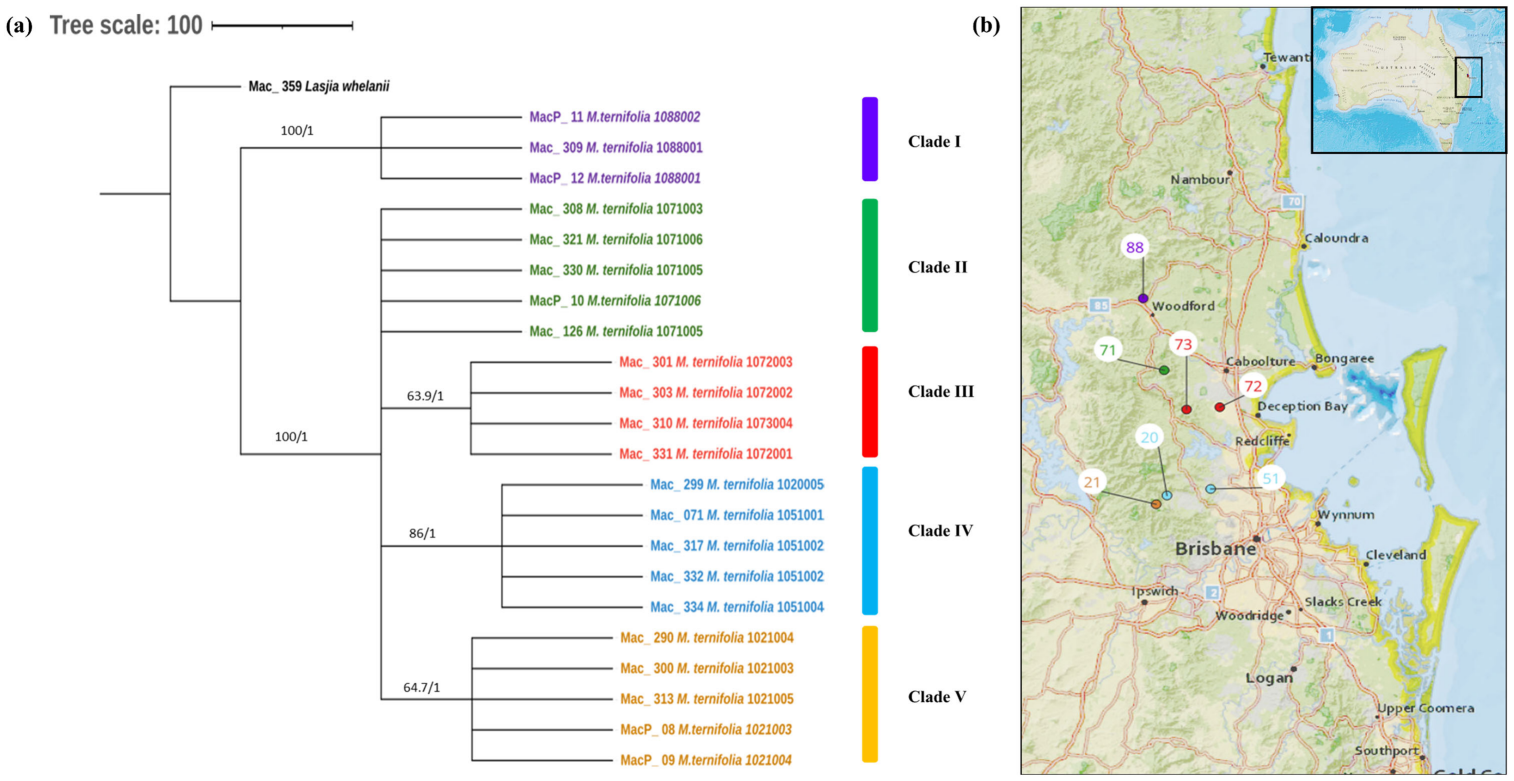
**(A)** Chloroplast phylogeny of *M. ternifolia* using the ML and BI method. **(B)** Phylogeographic analysis of *M. ternifolia*. Numbers above the lines represent ML bootstrap support/Bayesian posterior probabilities. Numbers indicate corresponding population site (**Supplementary Table 3**). Purple: Clade I, Green: Clade II, Red: Clade IIII, Light blue: Clade IV and Orange: Clade V. Coloured dots on the map indicate the corresponding clade in chloroplast phylogenetic tree.

The phylogenetic relationships within *M. ternifolia* accessions were inferred by 22 assembled complete chloroplast genomes. A multiple chloroplast alignment conducted using an outgroup was 160,807 bp with 97.2% identical sites. Phylogenetic trees built with the whole chloroplast genome using both methods had the same topology (**Figure 4A**). The results showed two major clades having MacP_11, Mac_309, and MacP_12 from Nambour (corresponding to population site 88) (**Figure 4B**) in one major clade and the remaining accessions in the second major clade. There was a clear relationship between the phylogenetic structure and geographic origin of the wild accessions of *M. ternifolia*. The resulting topology suggested accessions from Clade IV: Mac_299, Mac_071, Mac_317, Mac_332, and Mac_334 (corresponding to population sites 20, 51, 51, 51, and 51, respectively) were highly diverged accessions. In the *M. jansenii* population, no variants were observed, indicating that all accessions shared the same chloroplast haplotypes except for MacP_16 with a 2-bp difference.

**Figure 5:**
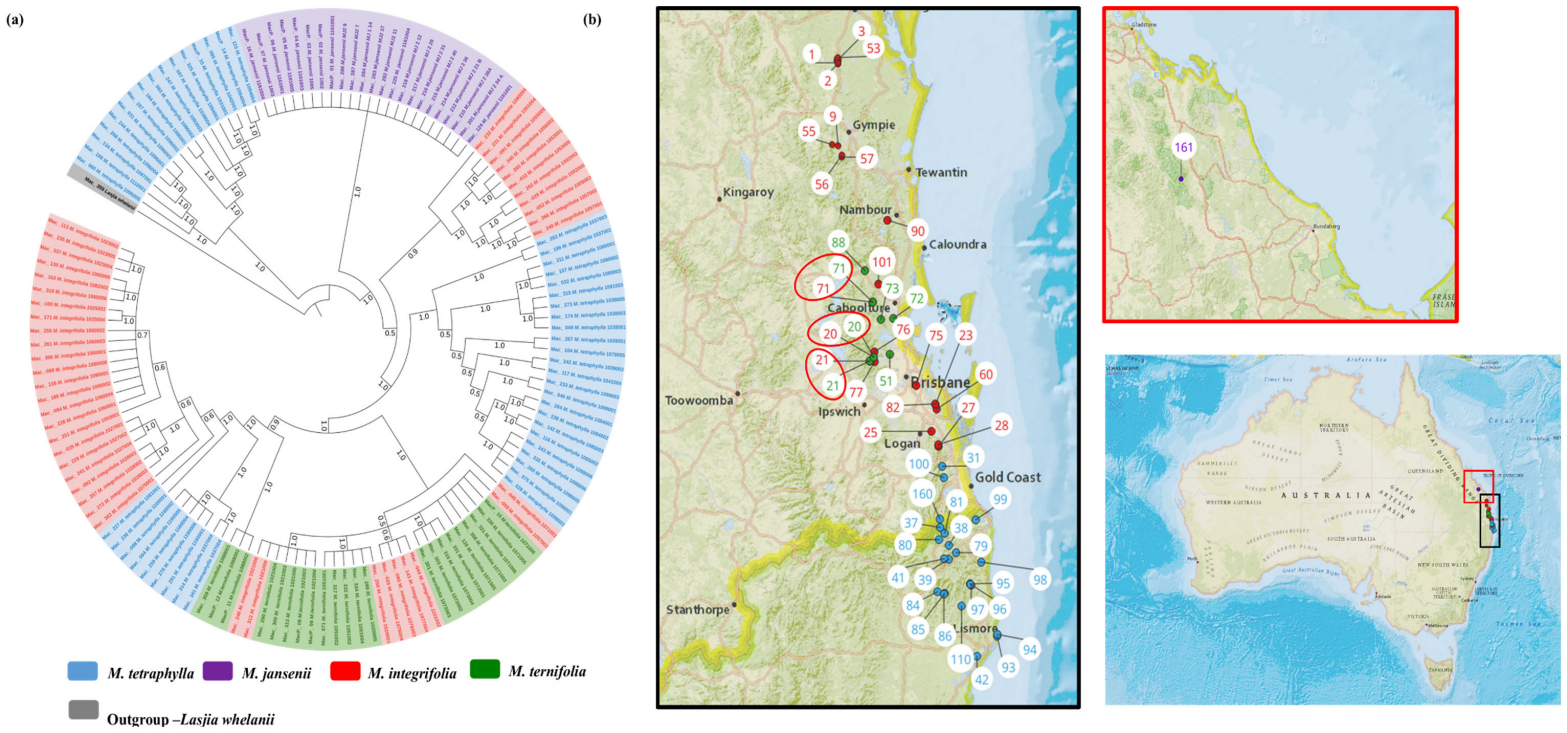
Chloroplast phylogeographic results of *Macadamia* species. **(A)** Chloroplast phylogenetic tree of Macadamia using BI method. Light blue: *M. tetraphylla*, Purple: *M. jansenii*, Red: *M. integrifolia* and Green: *M. ternifolia*. Numbers above the lines represent Bayesian posterior probabilities. **(B)** Map of Australia showing origins of Macadamia accessions. Numbers indicate corresponding population site (**Supplementary Table 3**). Coloured dots on the map indicate the corresponding species. Three red circles highlight population site number which contained accessions from different species.

The chloroplast phylogeny tree generated by taking 138 complete chloroplast genomes was supported with a PP of 1.0. Two major clades were identified (**Figure 5**). The first major clade contained 16 *M. tetraphylla* accessions from the Lismore region (Mac_060, Mac_108, Mac_134, Mac_268, Mac_244, Mac_031, Mac_297, Mac_184, Mac_083, and Mac_095), the Ballina region (Mac_247, Mac_325, MacP_14, and Mac_115), the Beenleigh region (MacP_15), and the Murwillumbah region (Mac_097). Interestingly, all these accessions corresponded to Clade I in *M. tetraphylla* chloroplast phylogenetic tree (**Figure 3A**). The second major clade was further differentiated into two sub-clades. All the *M. jansenii* accessions were clustered in one clade. The second sub-clade was further divided into two clades. The small sub-clade contained 10 accessions from the northern distribution of *M. integrifolia* (corresponding to Clade II in the *M. integrifolia* Cp phylogenetic tree) and 1 accession from the Nambour region (corresponding to Clade I in the *M. integrifolia* Cp phylogenetic tree). The larger sub-clade contained all the remaining *M. tetraphylla*, *M. integrifolia*, and *M. ternifolia.* This result shows that accessions that were collected from same locality cluster together. We assumed that chloroplast capture could be the reason for the presence of different species in the same clade when a species coexists in the same geographic area with other species.

### 3.3 Nuclear gene phylogenetic analysis

#### 3.3.1 Concatenation based phylogeny

For *M. integrifolia*, we used a total of 44 accessions. The multiple sequence alignment was 81,747 bp in length with 91% identical sites. The topology of the nuclear gene phylogenetic tree constructed based on both ML and BI methods was nearly identical (**Supplementary Figures 1**, **2**). However, the resulting phylogenetic trees exhibited low bootstrap values (<70) and Bayesian posterior probabilities (<0.95). Moreover, this result was not congruent with the results of the chloroplast phylogenetic tree.

The nucleotide alignment of 49 *M. tetraphylla* accessions along with the outgroup, was 81,753 bp in length. The phylogeny obtained with the ML approach was nearly identical to the BI approach (**Supplementary Figures 3**, **4**). Similar to *M. integrifolia* branching, the support rate is low. The tree topology of nuclear gene phylogeny and chloroplast phylogeny are dissimilar. Next, we constructed nuclear phylogenetic trees for *M. ternifolia* (**Supplementary Figures 5**, **6**) and *M. jansenii* (**Supplementary Figures 7**, **8**) populations with the ML and BI methods. However, the resulting topologies had poor statistical support for internal and external nodes.

**Figure 6:**
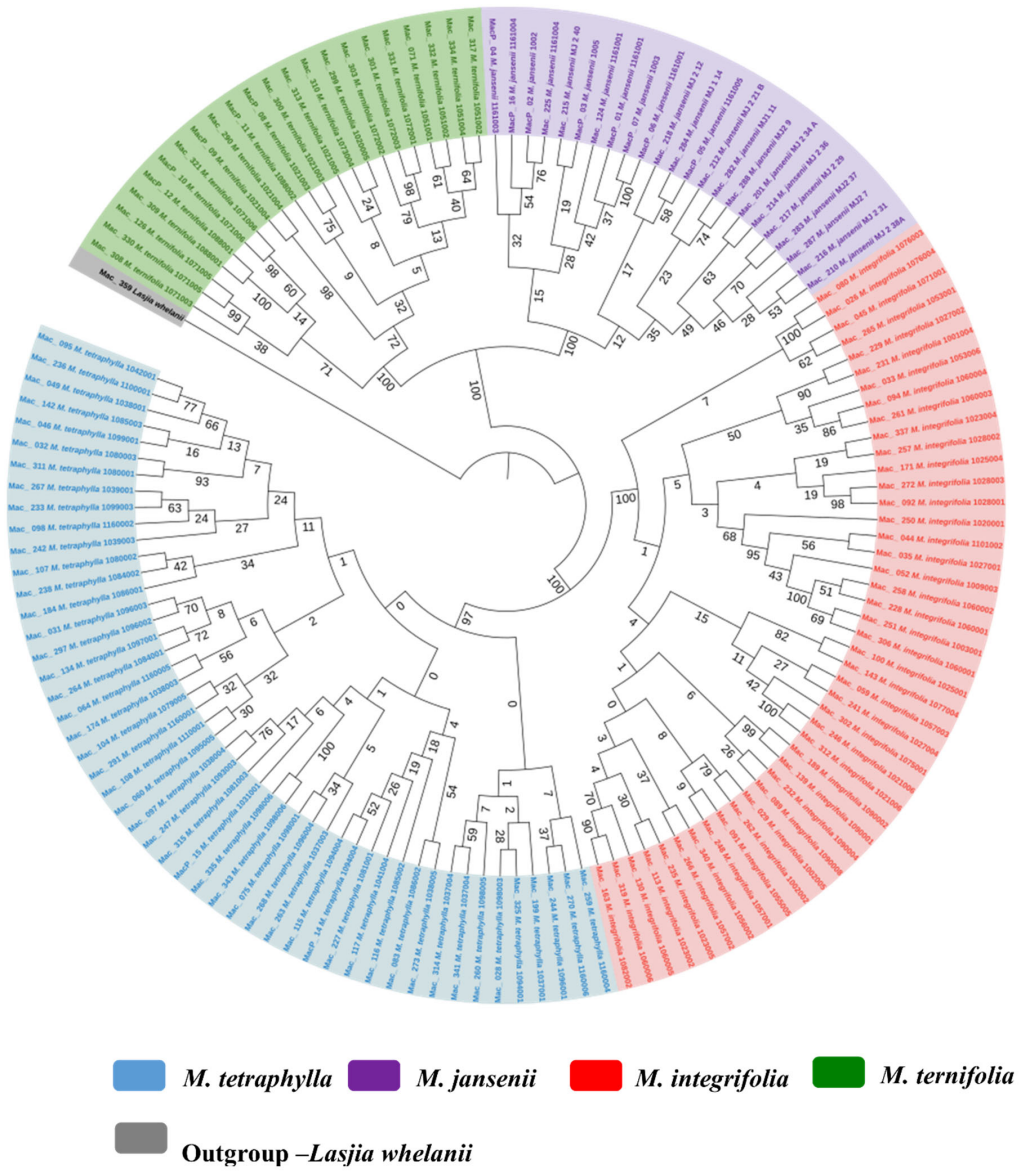
Nuclear phylogenetic results of *Macadamia* species. Light blue: *M. tetraphylla*, Purple: *M. jansenii*, Red: *M. integrifolia* and Green: *M. ternifolia*. Numbers above the lines represent ML bootstrap support. Phylogenetic tree constructed from coding sequences of 53 single copy genes using 1000 bootstrap replicates. Accessions were colour coded according to the species.

An ML tree was also constructed for 138 macadamia genotypes based on the concatenated single-copy nuclear gene CDS. The ML tree demonstrated the presence of four distinct species in the genus *Macadamia*. This well-supported tree classified the population into two main clades (**Figure 6**). The main Clade I consisted of *M. ternifolia* and *M. jansenii* while Clade II consisted of all *M. integrifolia* and *M. tetraphylla*. This outcome underscored the close relationship between *M. ternifolia* and *M. jansenii* and also that between *M. integrifolia* and *M. tetraphylla*.

#### 3.3.2 Single Nucleotide Polymorphisms (SNPs) based phylogeny

SNPs based phylogenetic tree for individual genes generated by talking 138 samples was supported with low bootstrap values (<70) (**Supplementary Figure 9**). However, the ASTRAL tree exhibited high local posterior probability support (LPP) values for external nodes (/1) (**Supplementary Figure 10**). This well-supported tree demonstrated *M. integrifolia* and *M. tetraphylla* in one clade and *M. ternifolia* and *M. jansenii* in another.

## 4 Discussion

Phylogenetics helps to unravel evolutionary histories and provides valuable insights into the factors driving the growth and adaptation of important plant groups worldwide (Lan et al., 2022). The current study, using whole-genome sequencing, has resolved the phylogeny of the four *Macadamia* species and confirmed that all the wild accessions belonged to four distinct species. Chloroplast genome sequences have been extensively used in Phylogenetic analysis (Sun et al., 2020). Chloroplasts are the most metabolically active organelle found in plants that carry out most of the biochemical synthesis process, which required the cell to produce energy through photosynthesis (Dobrogojski et al., 2020). The chloroplast DNA sequence has unique features compared to the nuclear genome in the analysis of population genetics and evolutionary relationships within families, genus, and species (De Las Rivas et al., 2002; Ahmad Termizi et al., 2016). The chloroplast genome is present in high copy numbers, has a low rate of spontaneous mutation, and does not undergo crossovers or recombination. Chloroplast genome sequence data are highly conserved (Dobrogojski et al., 2020). Earlier research relies on the separation of chloroplast genome from the nuclear genome and mitochondrial genome (Jansen et al., 2005). With the emergence of NGS technology, new high-throughput approaches have been introduced for the successful isolation of chloroplast sequencing with low cost (Ahmad Termizi et al., 2016). The read length, sequencing depth, sequence coverage or width, and evenness of coverage influence the accuracy of DNA sequencing using NGS technology. Short-read sequencing has been successfully applied to sequence chloroplast genomes of various plant species (Kyriakidou et al., 2018). Although there were numerous records of the extensive use of chloroplast genomes in evolutionary relationships in plants, very few studies were presented on the whole chloroplast genome data in macadamia. In this study, the size of all chloroplast genomes was consistent with previous macadamia chloroplast genomes (Nock et al., 2014; Liu et al., 2017, Liu et al., 2018). Genome annotation resulted in a higher number of genes compared to previous studies (Nock et al., 2014; Liu et al., 2017, Liu et al., 2018), which reported 79 CDS, 4 rRNA, and 30 tRNA for *M. integrifolia* (Nock et al., 2014), *M. ternifolia* (Liu et al., 2017), and *M. tetraphylla* (Liu et al., 2018). The difference in the gene number was possibly due to the difference in the annotation tool. All the previous genomes were annotated using Dual Organelle GenoMe Annotator (DOGMA) (Wyman et al., 2004), while the current genomes were annotated using the GeSeq online tool (https://chlorobox.mpimp-golm.mpg.de/geseq.html). Moreover, there is no previously reported chloroplast genome for *M. jansenii*. For the first time, we have generated chloroplast genomes for 23 *M. jansenii* accessions using the Get Organelle toolkit (Jin et al., 2020).

This study provides the most comprehensive analysis of the evolutionary relationships of the chloroplasts within the species in the genus *Macadamia*. The topology of the chloroplast phylogenetic tree with the distinct northern population and southern population of *M. integrifolia* is in agreement with the previously published phylogenetic results (Nock et al., 2019; Lin et al., 2022). Nock et al. (2019) also reported two distinct populations, namely, the Gundiah/Mount Bauple and the Gympie populations in the northern clade. A similar result was also reported by Lin et al. (2022) based on chloroplast and nuclear phylogenetic analysis. However, our results do not clearly separate accessions between the Gundiah/Mount Bauple region: Mac_231, Mac_262, Mac_029, Mac_265, and Mac_033 (corresponding to population sites 1, 2, 2, 3, and 3, respectively) (**Figure 2B**) and the Gympie region: Mac_052, Mac_091, Mac_340, Mac_248, and Mac_266 (corresponding to population sites 9, 55, 56, 57, and 57, respectively). In this study, the ML/BI phylogenetic tree showed that Mac_232 from Clade I (**Figure 2B**) was clustered separately from three other accessions in Cluster III (Mac_089, Mac_139, and Mac_189) originating from population site 90, suggesting that it is a planted tree. This finding was also supported by previous SSR results, which indicated that the Dulong tree is a planted tree that originated from the Brisbane region (Nock, 2022). The results also show that Mac_059 from population site 57 is a planted tree, which was not reported previously. Moreover, all the trees from the Sunshine Coast—Mac_089, Mac_139, and Mac_189 (**Figure 2A**, Clade IV)—are likely to be planted trees, which is consistent with previous studies (Nock et al., 2019; Nock, 2022). The results revealed that Mac_026, Mac_044, Mac_080, and Mac_143 are highly diverged accessions, in contrast to the previous study (Mai et al., 2020), in which Mac_229, Mac_266, Mac_139, and Mac_235 were recognized as diverged accessions. The phylogeographic results of the present study were in agreement with those of the previous study by Mai et al. (2020). Chloroplast phylogenetic analysis of *M. tetraphylla* revealed that MacP_15 (corresponding to population site 31), Mac_097 (corresponding to population site 38), and Mac_236 (corresponding to population site 100) (**Figures 3A, B**) might have been moved by humans as they were clustered with accessions from a different locality. It is noteworthy that we identified MacP_15 and Mac_236 to be outside the range of the natural population. *M. tetraphylla* is mostly distributed in the New South Wales region (Topp et al., 2019). There is no record of natural occurrence in the Beenleigh, QLD region. Previous studies reported a weak genetic differentiation (O'Connor et al., 2015; Mai et al., 2020; Nock, 2022) for *M. tetraphylla* populations. However, the present study revealed a positive correlation between genetics and geographical distribution.

For the first time, we report the phylogeographic pattern of distribution of genetic variation for the *M. ternifolia* population. However, further investigation is needed with an increased number of samples. Results for the *M. jansenii* accessions identified the presence of one chloroplast haplotype as expected due to the small, isolated population. This suggested that *M. jansenii* has gone through a genetic bottleneck. *M. jansenii* is found only in the Bulburin National Park north of Bundaberg, which is 180 km away from any *M. integrifolia* population (Topp et al., 2019; Mai et al., 2020). Therefore, the possibility of gene flow between the two populations is limited except for the movement of nuts with the involvement of humans. A decrease in the movement of genes is expected to increase the occurrence of inbreeding among individuals in the population (Hatmaker et al., 2018). Inbreeding, in turn, can have effects on the genetic health of the population, potentially leading to an accumulation of harmful traits and a decrease in overall fitness (Hatmaker et al., 2018).

In contrast to the previously recorded phylogenies (Peace, 2005; Mast et al., 2008; Mai et al., 2020; Nock, 2022), we found that accessions that were collected from the same geographical location were closely related. The distinct separation of *Macadamia* populations within species reported in previous phylogenies (Peace, 2005; Mast et al., 2008; Mai et al., 2020; Nock, 2022) were based on chloroplast genome analysis. This study shows that reticulate evolution has resulted in chloroplast transfer between species and resulted in distinct chloroplast types within individual species but does not affect the distinctness of the nuclear genomes. Although chloroplast capture was not previously reported in Macadamia, many other plants have reported the occurrence of reticulate evolution of the chloroplast (Acosta and Premoli, 2010; Wambugu et al., 2015; Yi et al., 2015; Moner et al., 2020; Ananda et al., 2021). In this study, chloroplast phylogeny separated 16 *M. tetraphylla* accessions from the rest of the accessions. The second major clade was further divided into two clades having all *M. jansenii* in one clade and the rest of the accessions in the other. Furthermore, a sub-clade further separated 11 *M. integrifolia* and 24 *M. tetraphylla* accessions, leaving a complex clade having *M. integrifolia*, *M. ternifolia*, and *M. tetraphylla*. This suggested a series of chloroplast capture events between *M. integrifolia*, *M. ternifolia*, and *M. tetraphylla.*

Phylogenetic trees built with the single-copy nuclear gene CDS for *Macadamia* species strongly supported four distinct species in the genus *Macadamia* as reported in previous studies (Peace, 2005; Nock, 2022). Individual nuclear phylogenetic trees for the four species showed little structure, suggesting widespread gene flow within each species and little geographic structure in the nuclear genome. The SNPs based phylogeny also showed four distinct species in the genus *Macadamia*.

The availability of large sequence data has significantly advanced our understanding of the distribution and diversity of *Macadamia* species, which is essential for both conservation and breeding programs. This advanced knowledge aids in the conservation of these species, now found in fragmented rainforest habitats, by highlighting the importance of *in situ* conservation strategies that focus on capturing a wide range of genetic diversity within sites. Such conservation efforts are crucial not only for safeguarding the species against extinction but also in enhancing their commercial value and sustainability for the future generations.

## Data Availability

All sequence data available at NCBI via Bio Project: PRJNA1036028 with Bio sample number SAMN38356272 - SAMN38356438 (**Supplementary Table 8**).
