# Supplementary material for: Analysis of phylogenetic relationships in Macadamia shows evidence of extensive reticulate evolution
Source: Front Plant Sci. 2024 Oct 15;15:1394244. doi: 10.3389/fpls.2024.1394244 (PMC11518779; doi:10.3389/fpls.2024.1394244)
Supplement: Supplementary file 1 [file DataSheet1.docx]

**Supplemental Information for:**

**Analysis of phylogenetic relationships in *Macadamia* shows evidence of extensive reticulate evolution**

Sachini Lakmini Manatunga^1,2^, Agnelo Furtado^1^, Bruce Topp^3^, Mobashwer Alam^3^, Patrick J. Mason^1,2^, Ardashir Kharabian-Masouleh^1,2^, Robert J Henry^1,2^*

^1^Queensland Alliance for Agriculture & Food Innovation (QAAFI), University of Queensland, St Lucia QLD 4072, Australia, The University of Queensland, Carmody Rd, St Lucia QLD 4072

^2^ARC Centre of Excellence for Plant Success in Nature and Agriculture, The University of Queensland, Carmody Rd, St Lucia QLD 4072

^3^Queensland Alliance for Agriculture & Food Innovation (QAAFI), The University of Queensland, Maroochy Research Facility, Nambour QLD 4560

*Corresponding authors: robert.henry@uq.edu.au


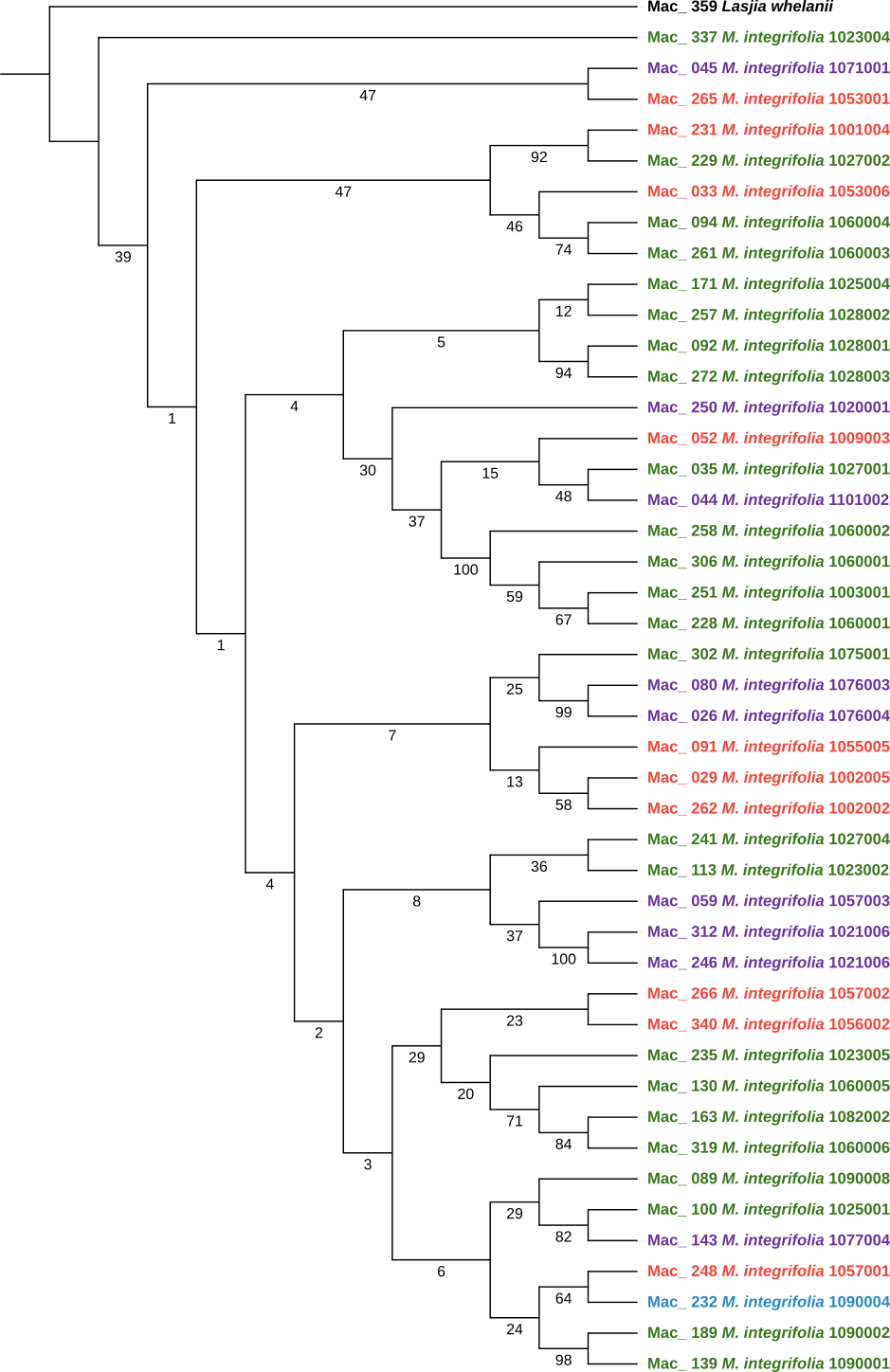


**Supplementary figure 1: Nuclear gene phylogeny of *M. integrifolia* using Maximum likelihood (ML) method.** Numbers above the lines represent ML bootstrap support value. Phylogenetic tree constructed from coding sequences of 53 single copy genes using 1000 bootstrap replicates. Accessions were color coded according to the chloroplast phylogenetic clade separation.


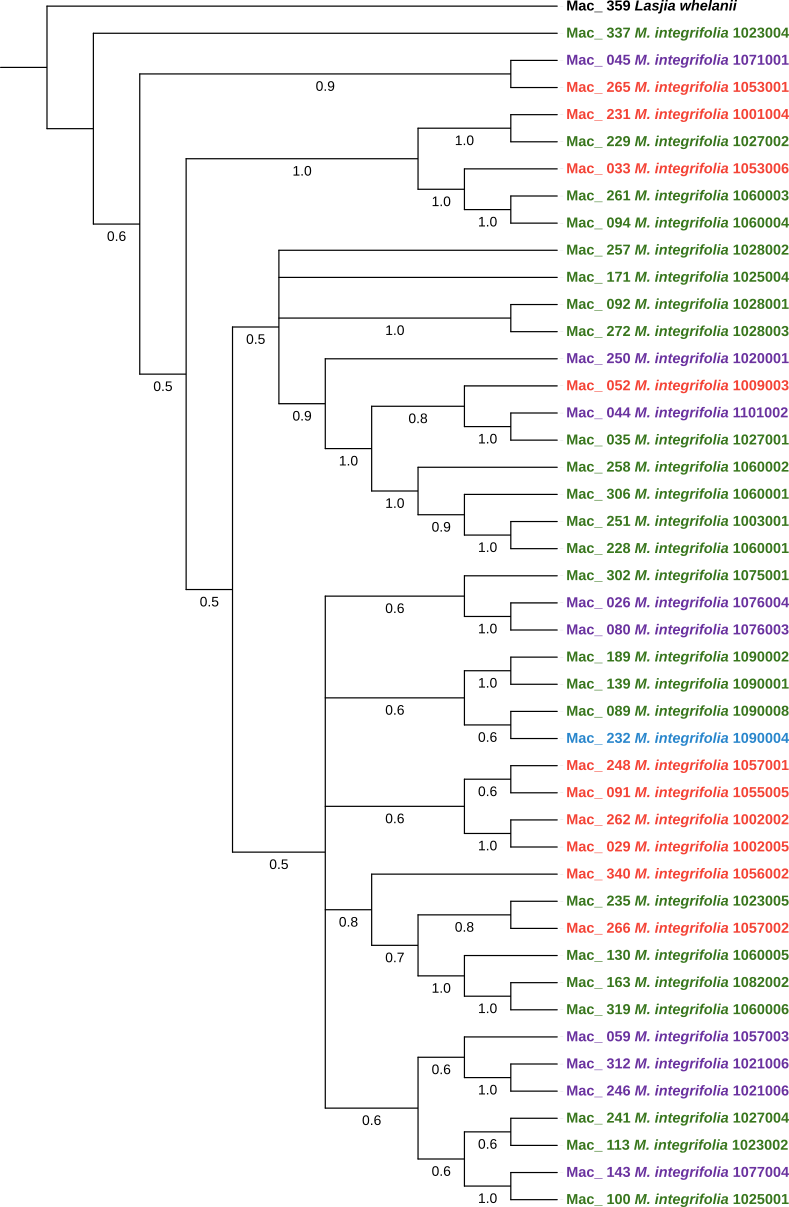


**Supplementary figure 2: Nuclear gene phylogeny of *M. integrifolia* using Bayesian inference (BI) method.** Numbers above the lines represent Bayesian posterior probabilities. Phylogenetic tree constructed from coding sequences of 53 single copy genes. Accessions were color coded according to the chloroplast phylogenetic clade separation.


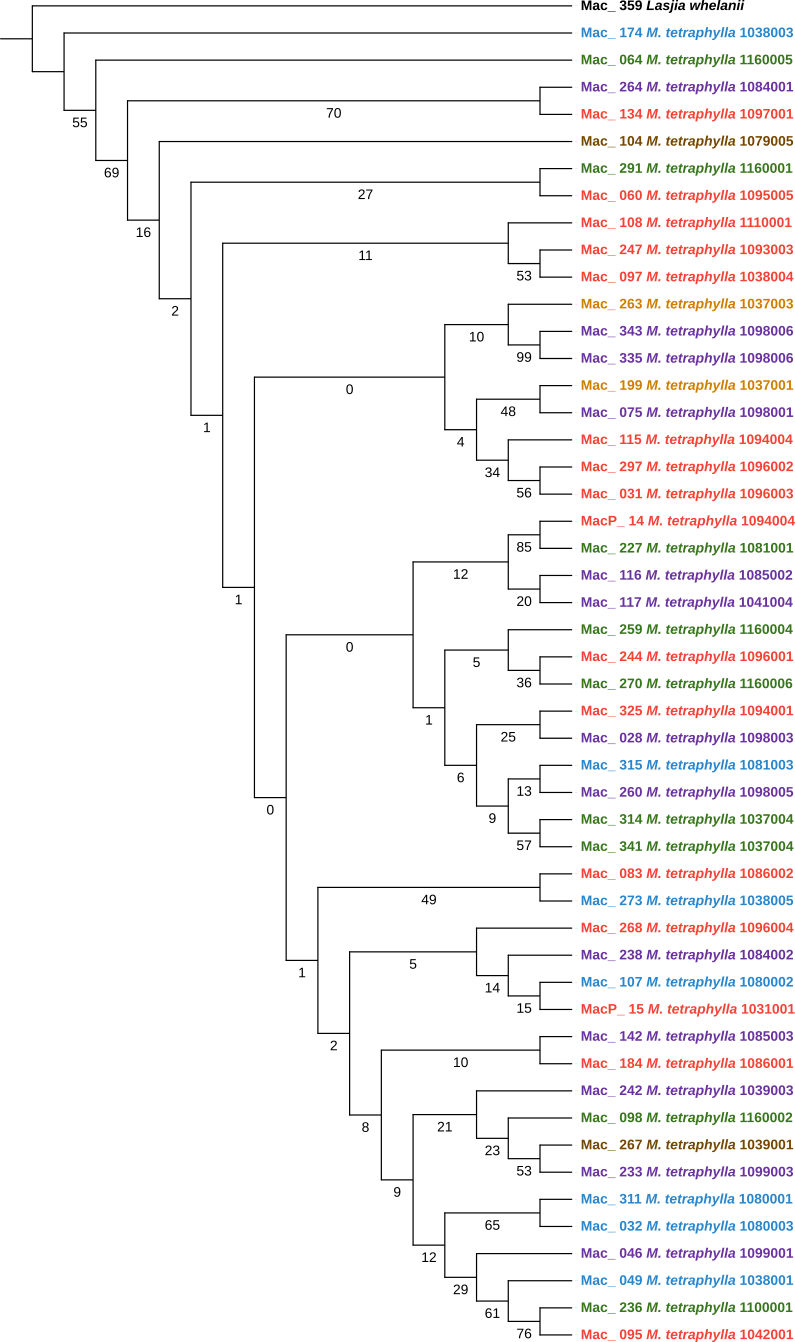


**Supplementary figure 3: Nuclear gene phylogeny of *M. tetraphylla* using Maximum likelihood (ML) method.** Numbers above the lines represent ML bootstrap support value. Phylogenetic tree constructed from coding sequences of 53 single copy genes using 1000 bootstrap replicates. Accessions were color coded according to the chloroplast phylogenetic clade separation.


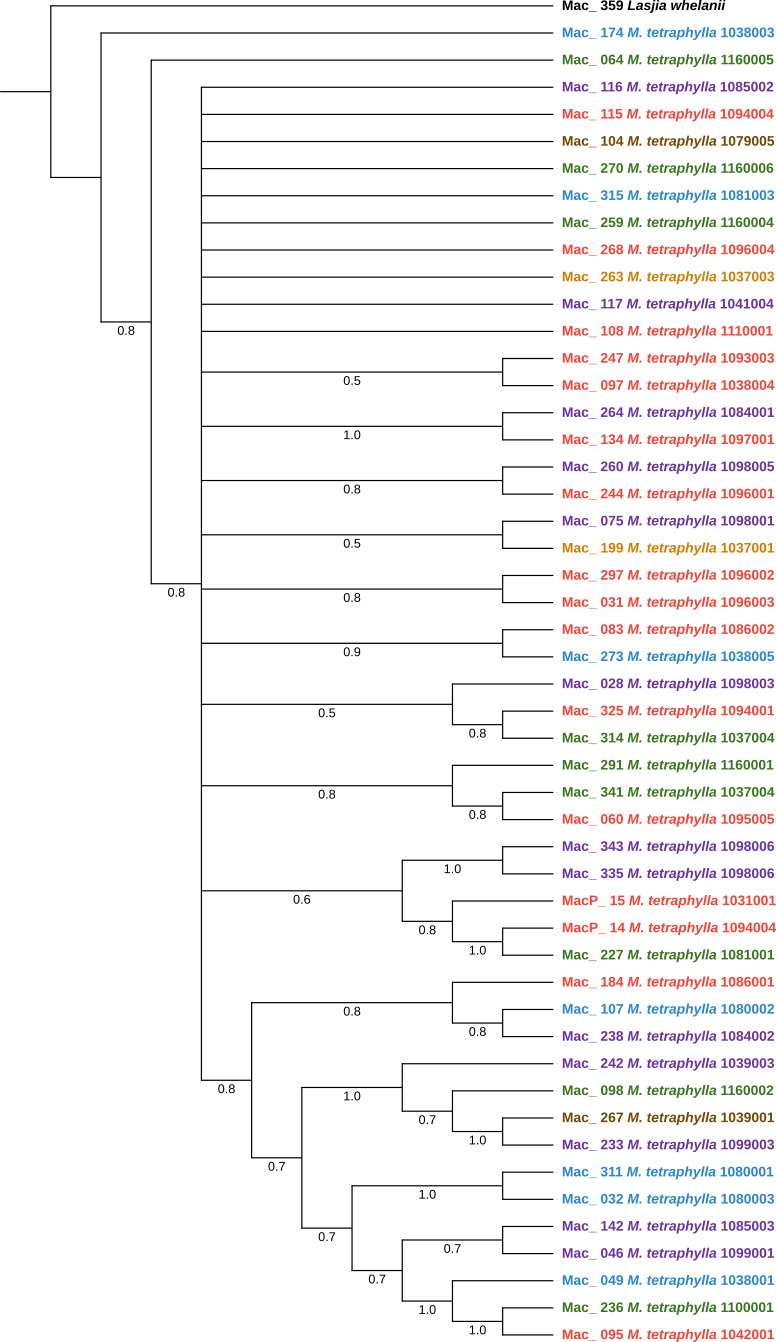


**Supplementary figure 4: Nuclear gene phylogeny of *M. tetraphylla* using Bayesian inference (BI) method.** Numbers above the lines represent Bayesian posterior probabilities. Phylogenetic tree constructed from coding sequences of 53 single copy genes. Accessions were color coded according to the chloroplast phylogenetic clade separation.


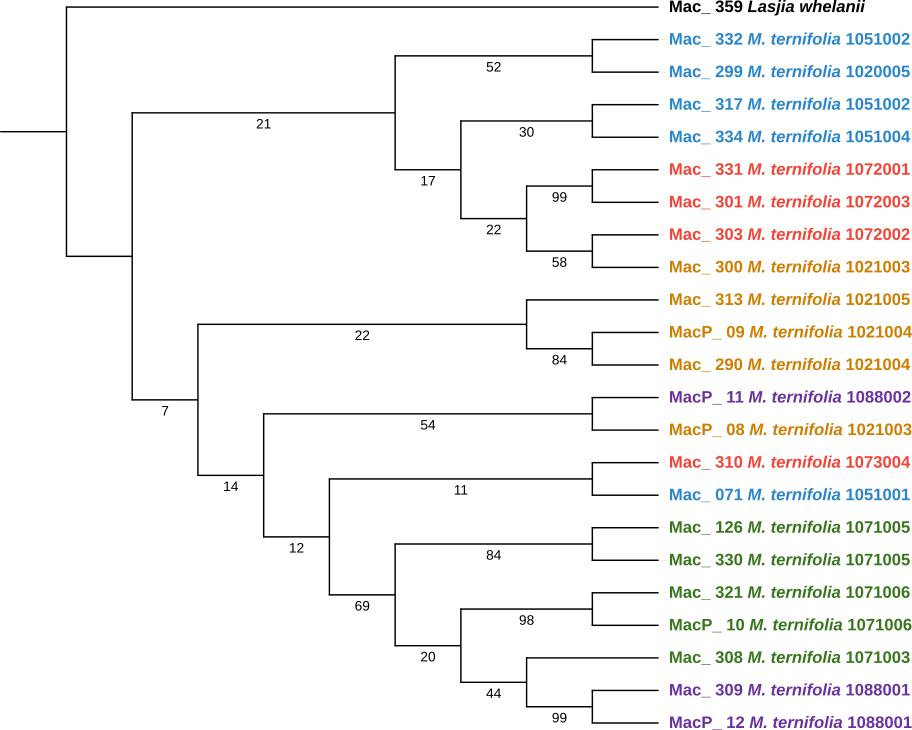


**Supplementary figure 5: Nuclear gene phylogeny of *M. ternifolia* using Maximum likelihood (ML) method.** Numbers above the lines represent ML bootstrap support value. Phylogenetic tree constructed from coding sequences of 53 single copy genes using 1000 bootstrap replicates. Accessions were color coded according to the chloroplast phylogenetic clade separation.


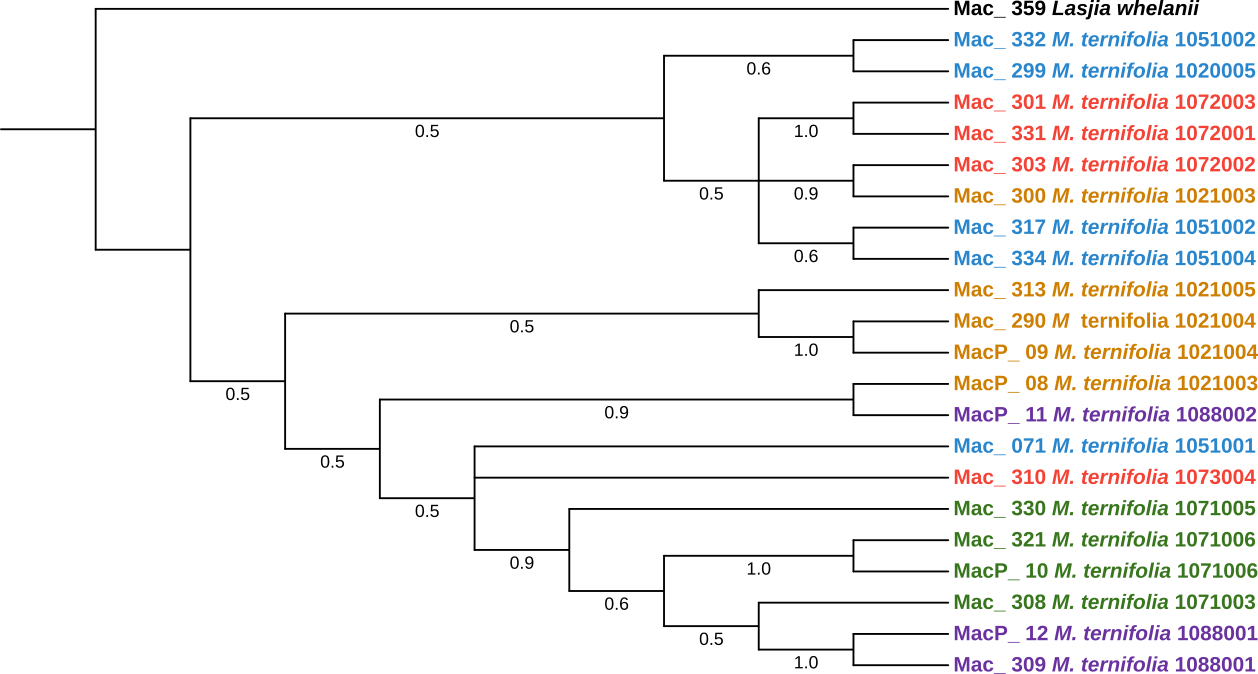


**Supplementary figure 6: Nuclear gene phylogeny of *M. ternifolia* using Bayesian inference (BI) method.** Numbers above the lines represent Bayesian posterior probabilities. Phylogenetic tree constructed from coding sequences of 53 single copy genes. Accessions were color coded according to the chloroplast phylogenetic clade separation.


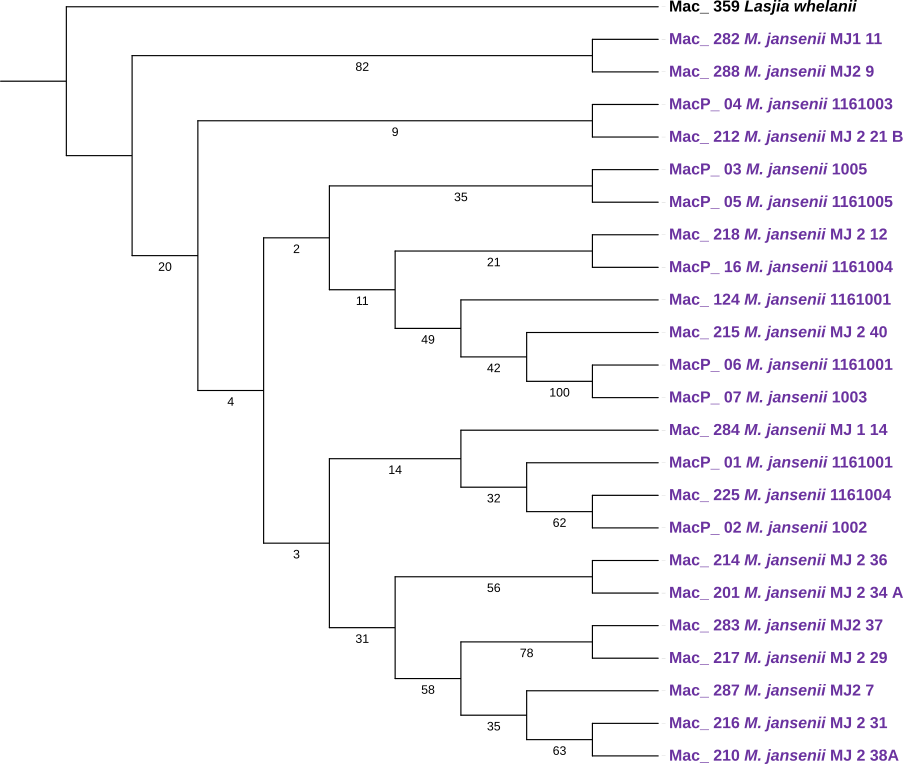


**Supplementary figure 7: Nuclear gene phylogeny of *M. jansenii* using Maximum likelihood (ML) method.** Numbers above the lines represent ML bootstrap support value. Phylogenetic tree constructed from coding sequences of 53 single copy genes using 1000 bootstrap replicates. Accessions were color coded according to the chloroplast phylogenetic clade separation.


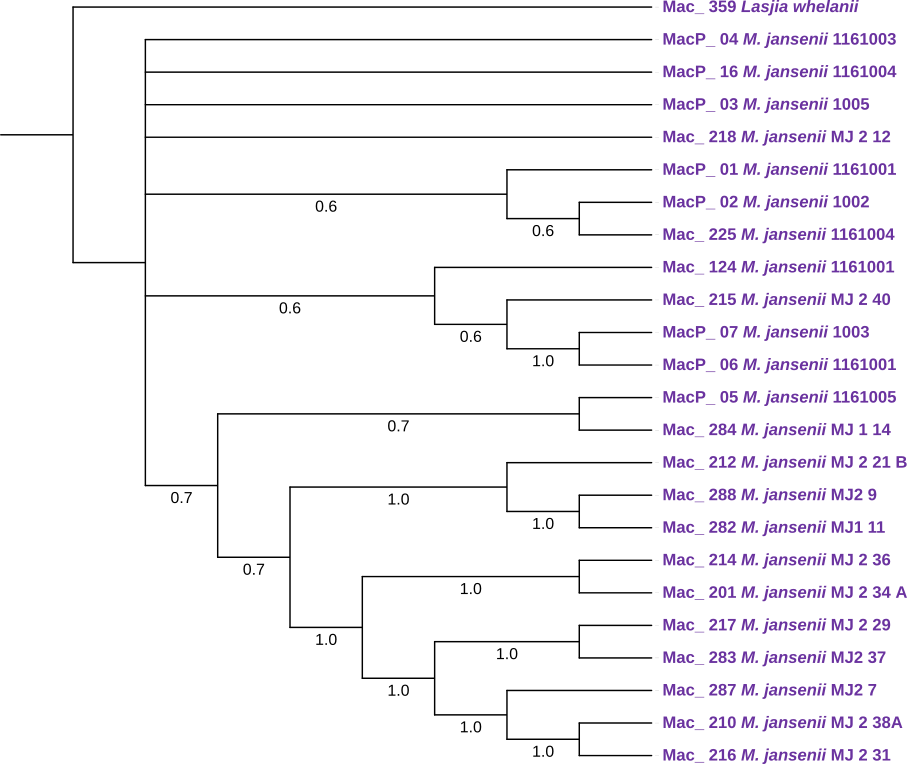


**Supplementary figure 8: Nuclear gene phylogeny of *M. jansenii* using Bayesian inference (BI) method.** Numbers above the lines represent Bayesian posterior probabilities. Phylogenetic tree constructed from coding sequences of 53 single copy genes. Accessions were color coded according to the chloroplast phylogenetic clade separation.
