# Supplementary material for: Analysis of phylogenetic relationships in Macadamia shows evidence of extensive reticulate evolution
Source: Front Plant Sci. 2024 Oct 15;15:1394244. doi: 10.3389/fpls.2024.1394244 (PMC11518779; doi:10.3389/fpls.2024.1394244)
Supplement: Supplementary file 2 [file DataSheet2.docx]

**Supplemental Information for:**

**Analysis of phylogenetic relationships in *Macadamia* shows evidence of extensive reticulate evolution**

Sachini Lakmini Manatunga^1,2^, Agnelo Furtado^1^, Bruce Topp^3^, Mobashwer Alam^3^, Patrick J. Mason^1,2^, Ardashir Kharabian-Masouleh^1,2^, Robert J Henry^1,2^*

^1^Queensland Alliance for Agriculture & Food Innovation (QAAFI), University of Queensland, St Lucia QLD 4072, Australia, The University of Queensland, Carmody Rd, St Lucia QLD 4072

^2^ARC Centre of Excellence for Plant Success in Nature and Agriculture, The University of Queensland, Carmody Rd, St Lucia QLD 4072

^3^Queensland Alliance for Agriculture & Food Innovation (QAAFI), The University of Queensland, Maroochy Research Facility, Nambour QLD 4560

*Corresponding authors: robert.henry@uq.edu.au

**
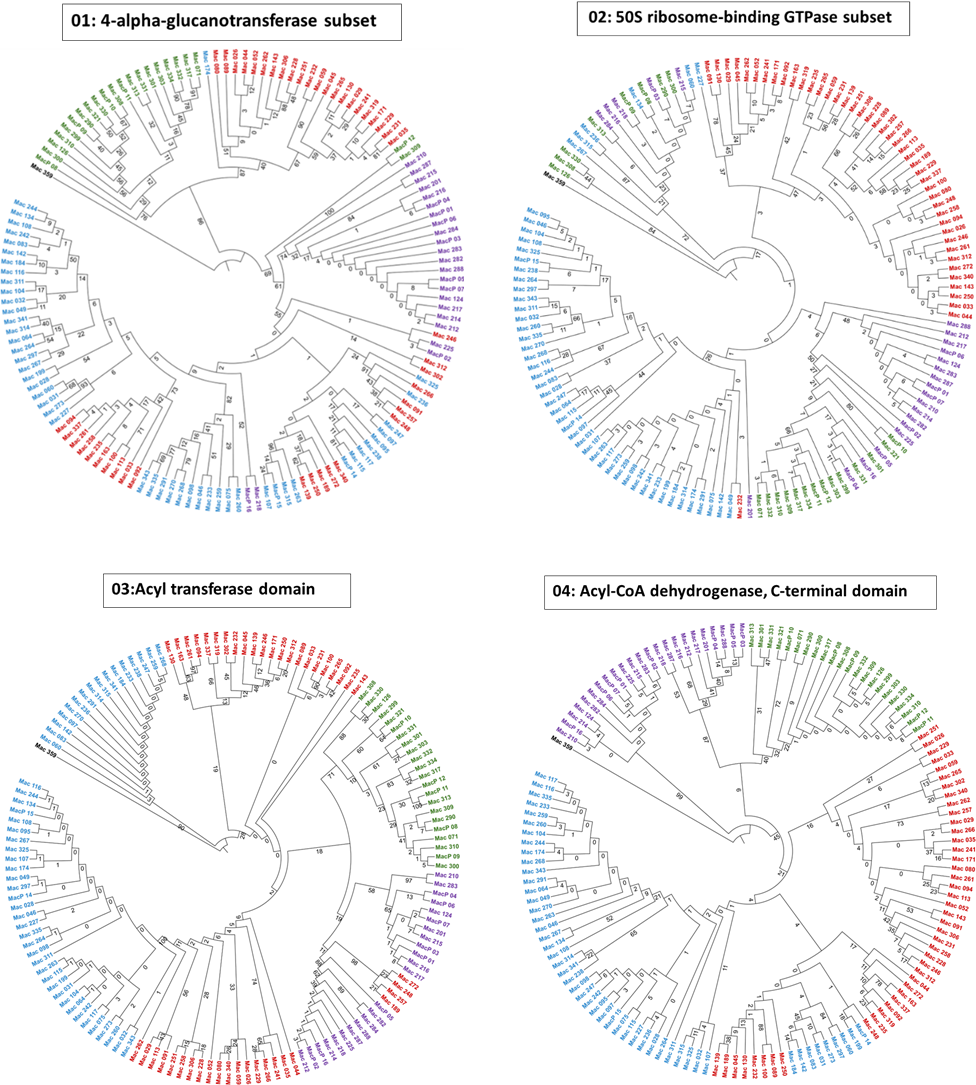
**

**Figure 9: Individual gene trees constructed for 45 nuclear gene CDS sequences (Gene trees 1-4)**. Light blue: *M. tetraphylla*, Purple: *M. jansenii*, Red: *M. integrifolia*, Green: *M. ternifolia and Black: L. whelanii*. Numbers above the lines represent ML bootstrap support. Phylogenetic tree constructed using 1000 bootstrap replicates. Accessions were colour coded according to the species.


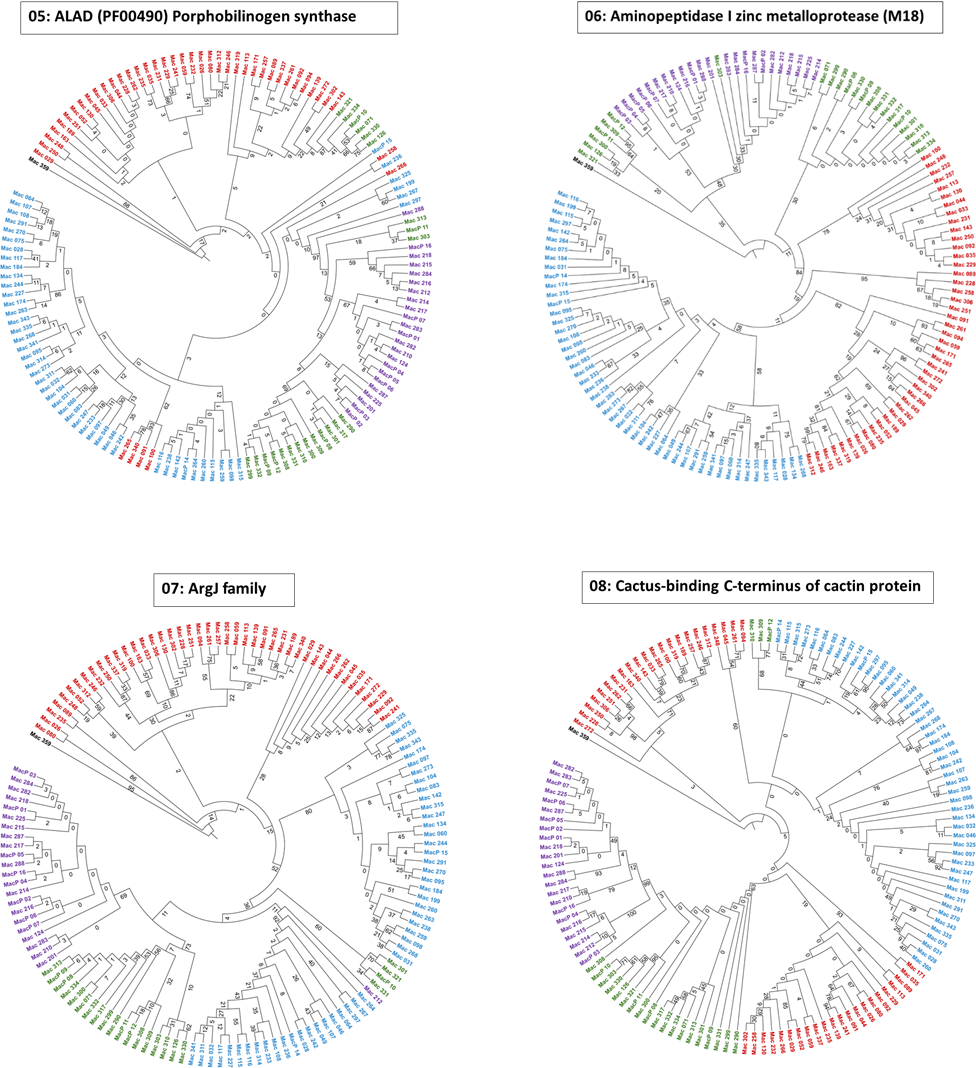


**Figure 9: Individual gene trees constructed for 45 nuclear gene CDS sequences (Gene trees 5-8)**. Light blue: *M. tetraphylla*, Purple: *M. jansenii*, Red: *M. integrifolia,* Green: *M. ternifolia and Black: L. whelanii*. Numbers above the lines represent ML bootstrap support. Phylogenetic tree constructed using 1000 bootstrap replicates. Accessions were colour coded according to the species.


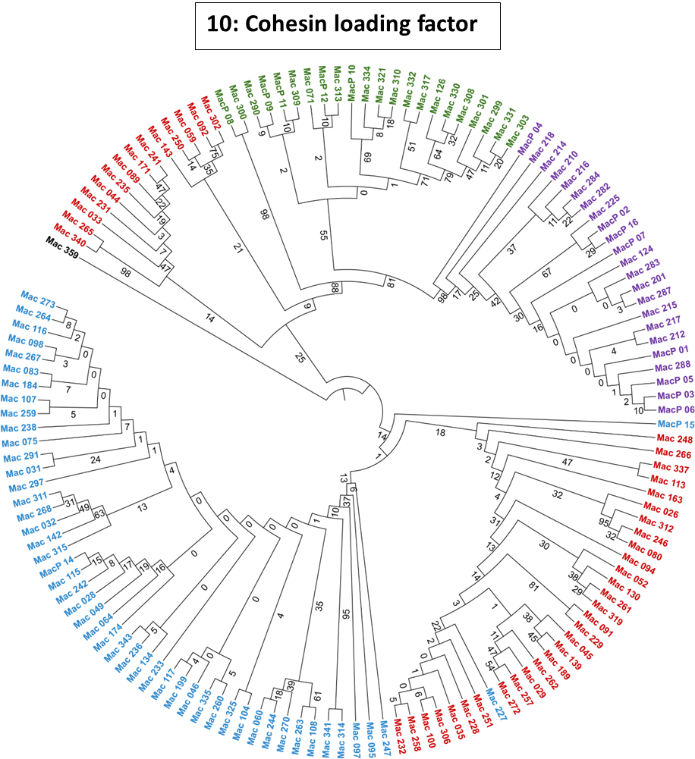

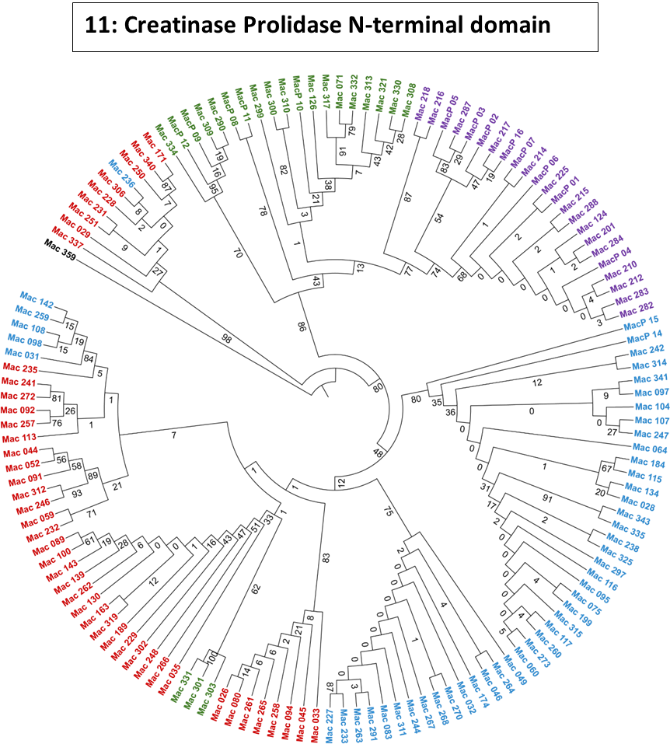

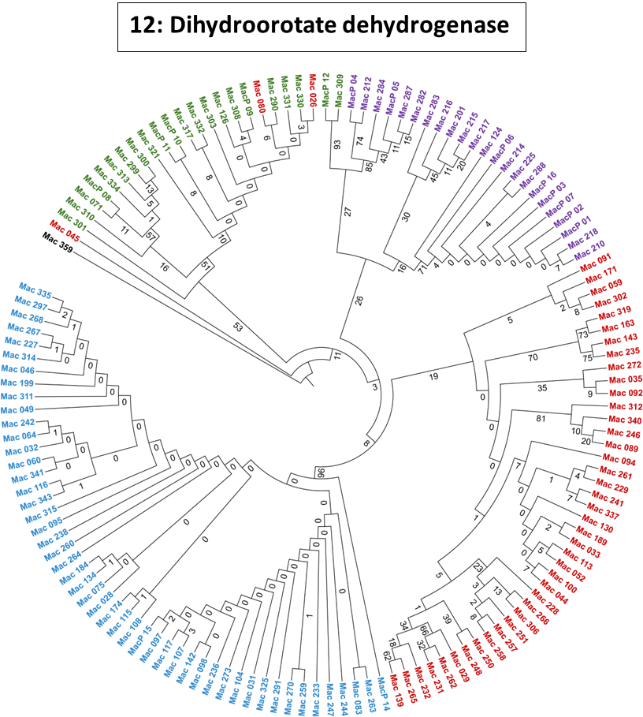

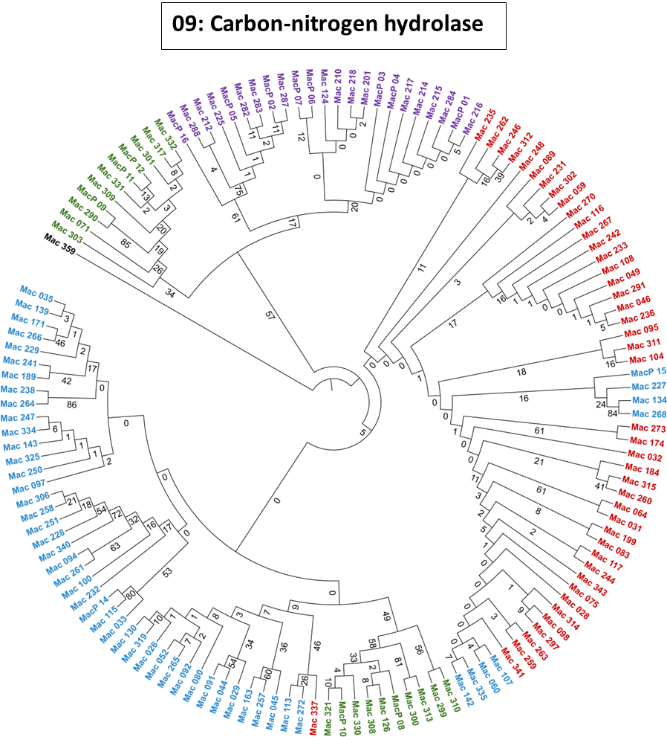


**Figure 9: Individual gene trees constructed for 45 nuclear gene CDS sequences (Gene trees 9-12)**. Light blue: *M. tetraphylla*, Purple: *M. jansenii*, Red: *M. integrifolia,* Green: *M. ternifolia and Black: L. whelanii.* Numbers above the lines represent ML bootstrap support. Phylogenetic tree constructed using 1000 bootstrap replicates. Accessions were colour coded according to the species.


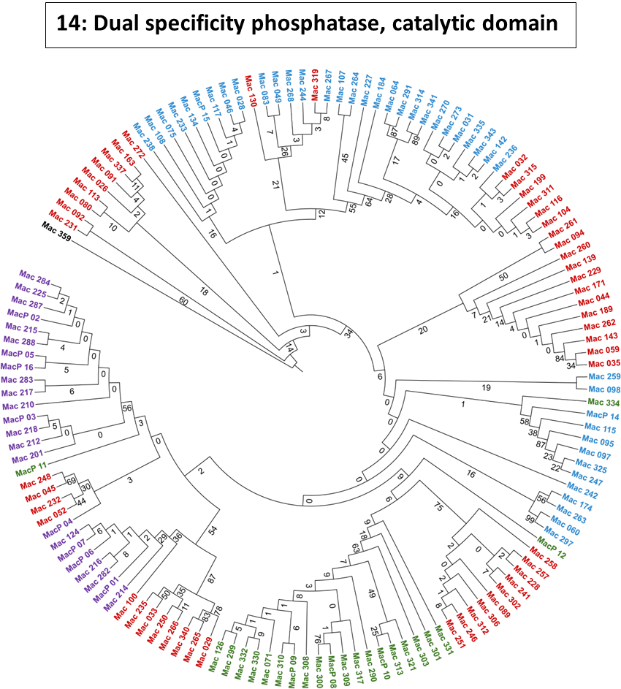

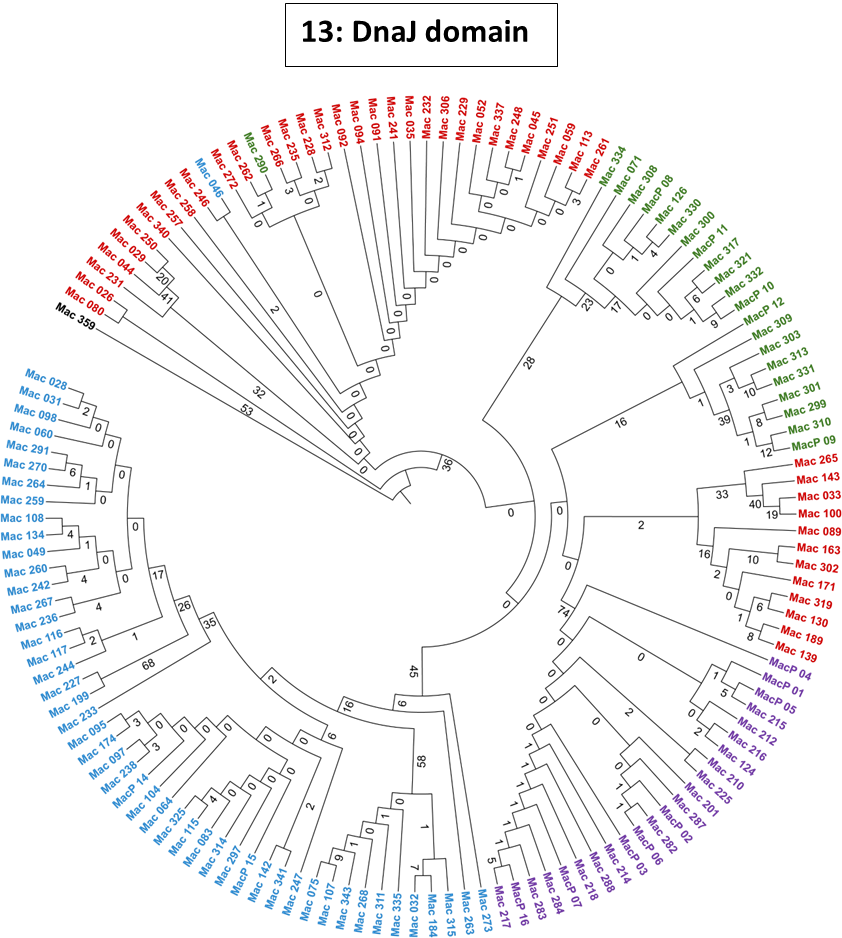

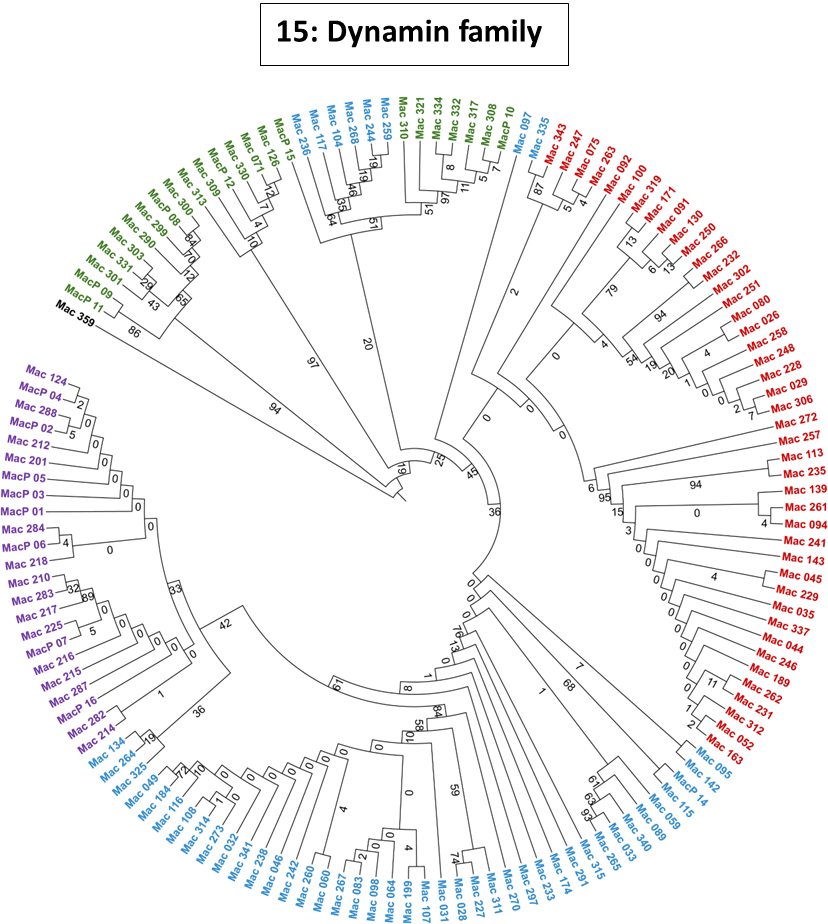

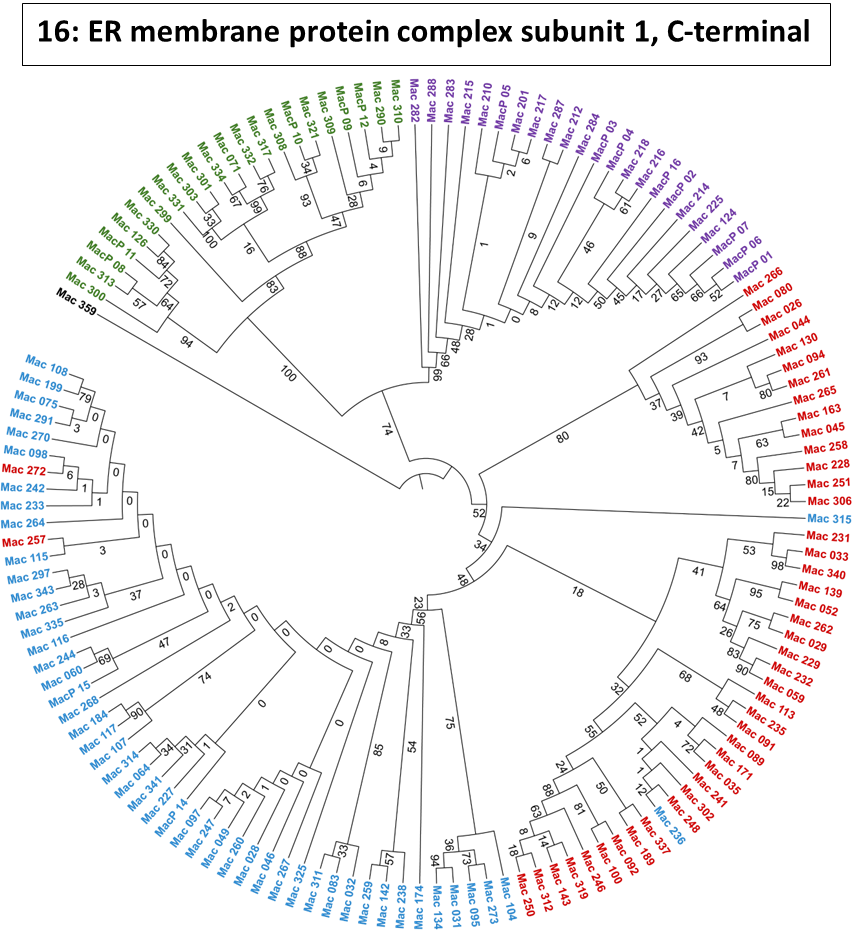


**Figure 9: Individual gene trees constructed for 45 nuclear gene CDS sequences (Gene trees 13-16)**. Light blue: *M. tetraphylla*, Purple: *M. jansenii*, Red: *M. integrifolia,* Green: *M. ternifolia and Black: L. whelanii*. Numbers above the lines represent ML bootstrap support. Phylogenetic tree constructed using 1000 bootstrap replicates. Accessions were colour coded according to the species.


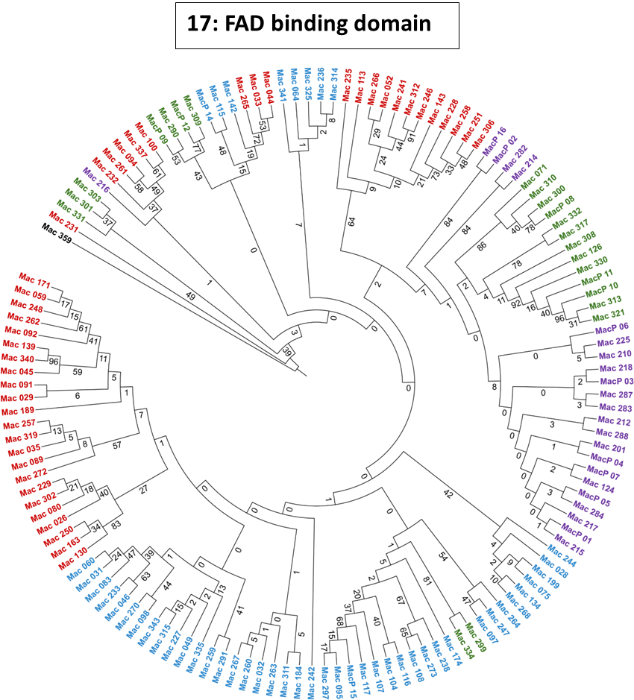

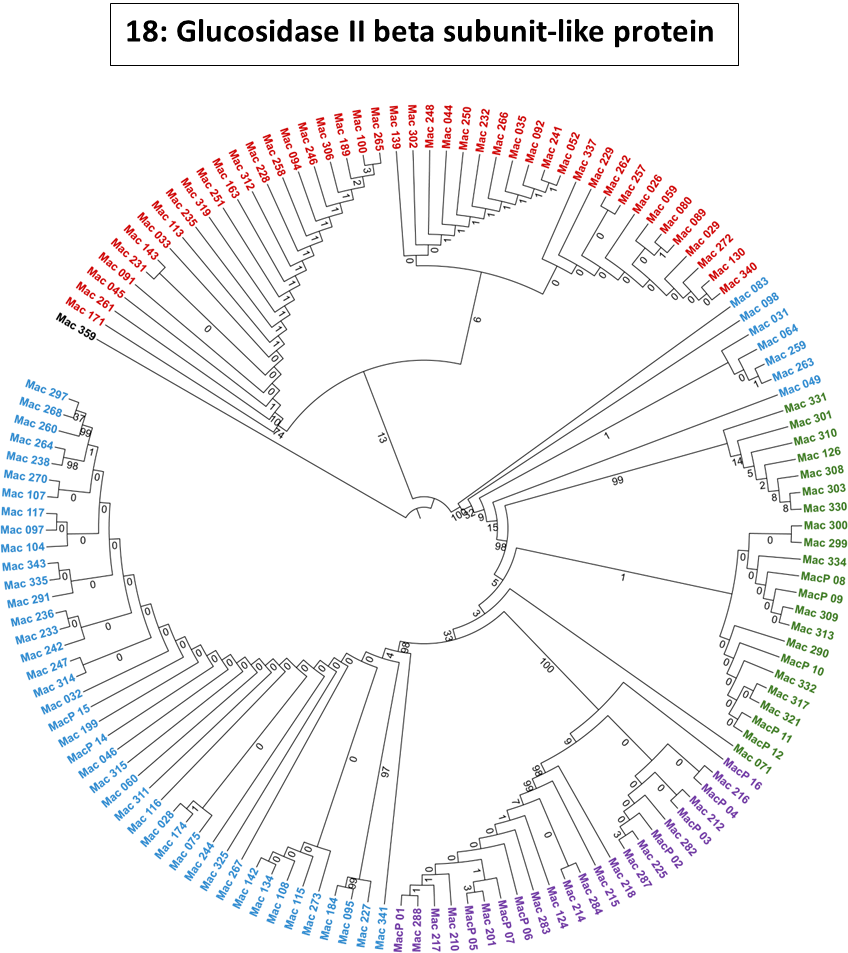

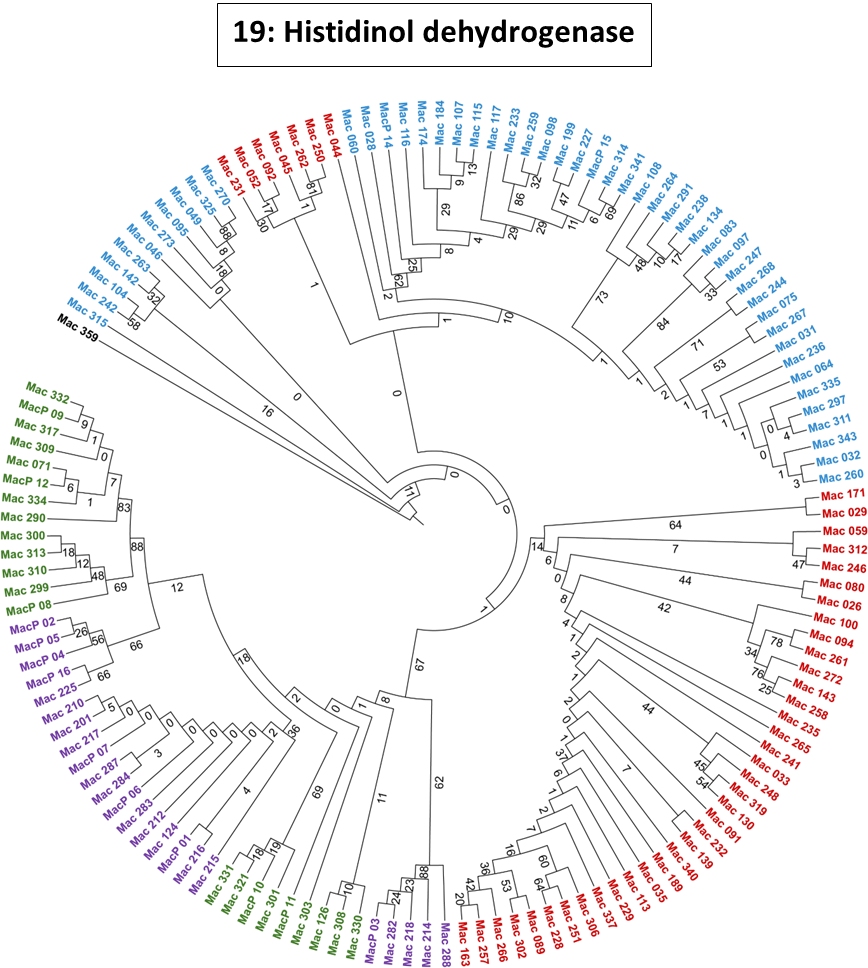

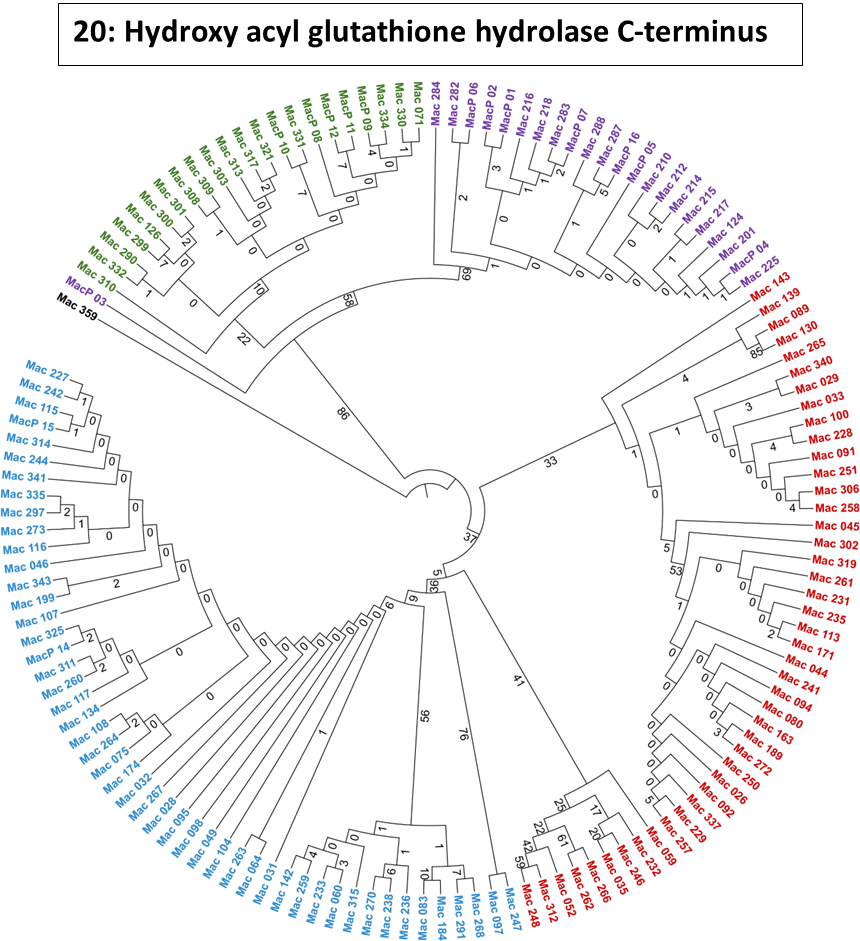


**Figure 9: Individual gene trees constructed for 45 nuclear gene CDS sequences (Gene trees 17-20)**. Light blue: *M. tetraphylla*, Purple: *M. jansenii*, Red: *M. integrifolia,* Green: *M. ternifolia and Black: L. whelanii*. Numbers above the lines represent ML bootstrap support. Phylogenetic tree constructed using 1000 bootstrap replicates. Accessions were colour coded according to the species.


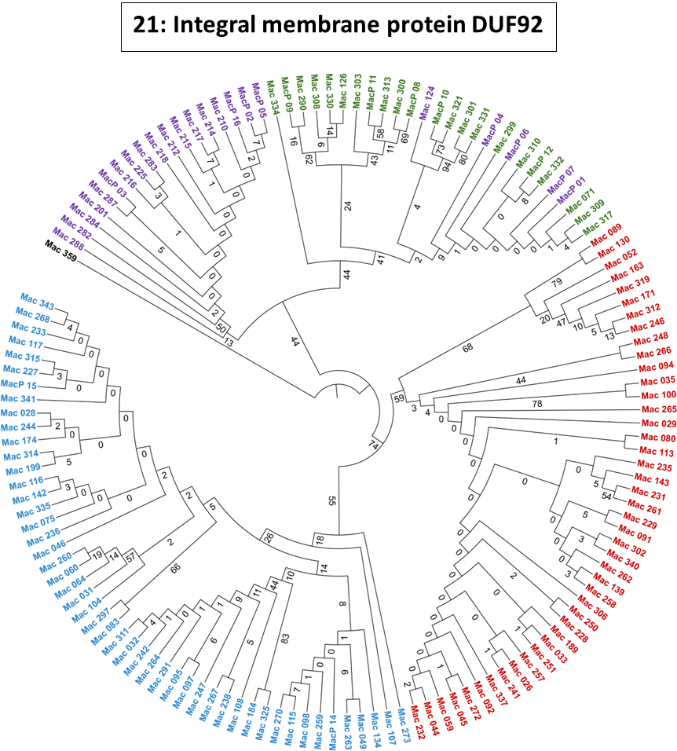

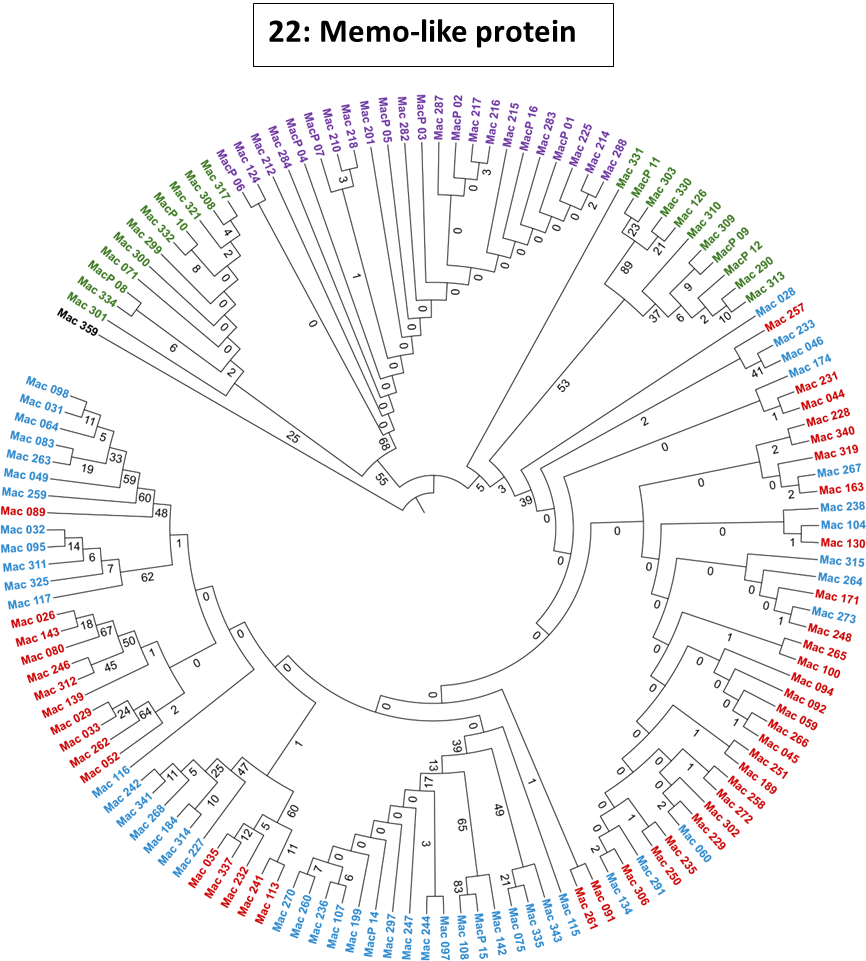

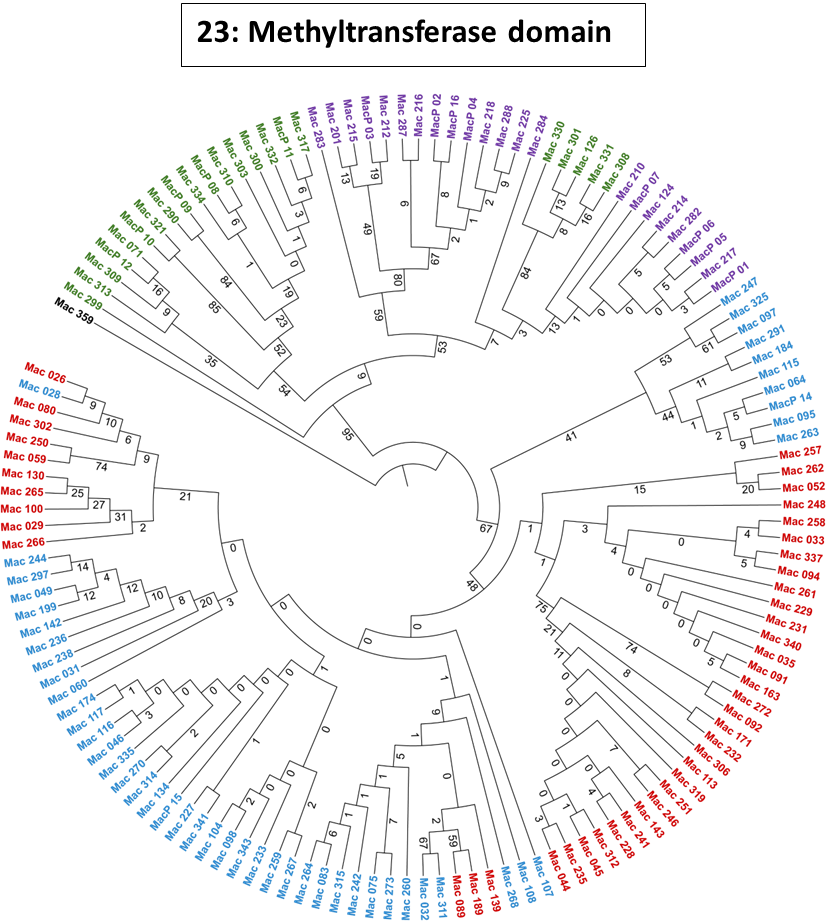

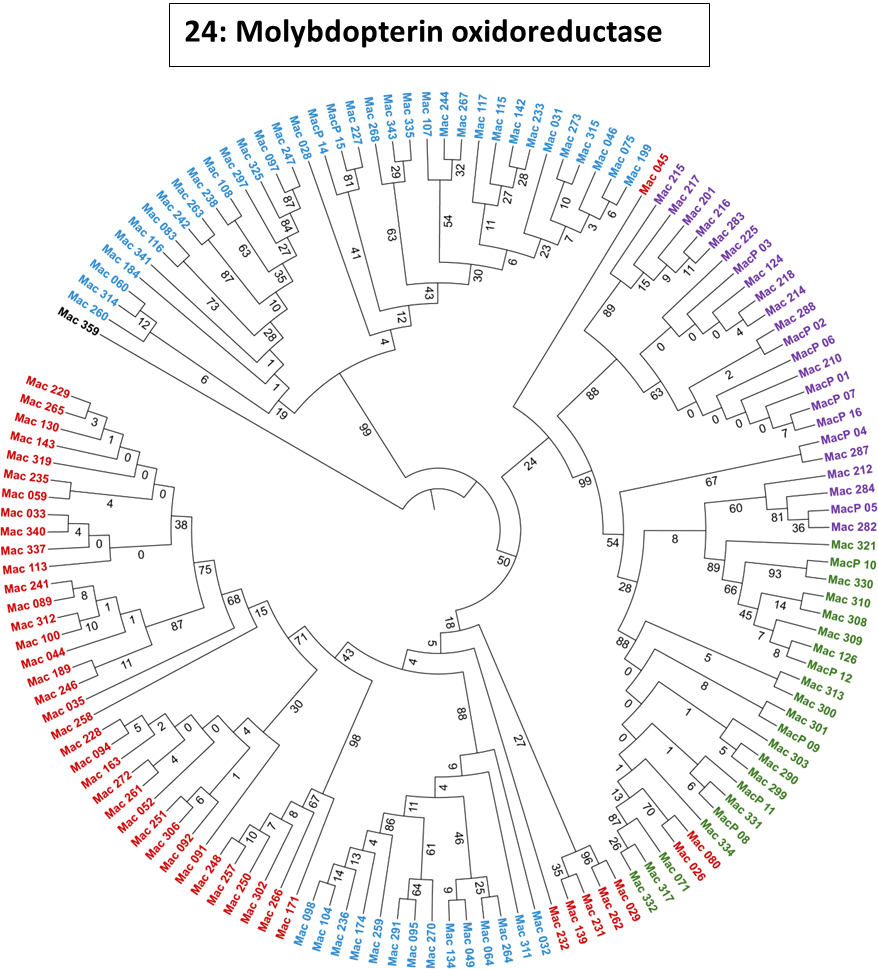


**Figure 9: Individual gene trees constructed for 45 nuclear gene CDS sequences (Gene trees 21-24)**. Light blue: *M. tetraphylla*, Purple: *M. jansenii*, Red: *M. integrifolia,* Green: *M. ternifolia and Black: L. whelanii*. Numbers above the lines represent ML bootstrap support. Phylogenetic tree constructed using 1000 bootstrap replicates. Accessions were colour coded according to the species.


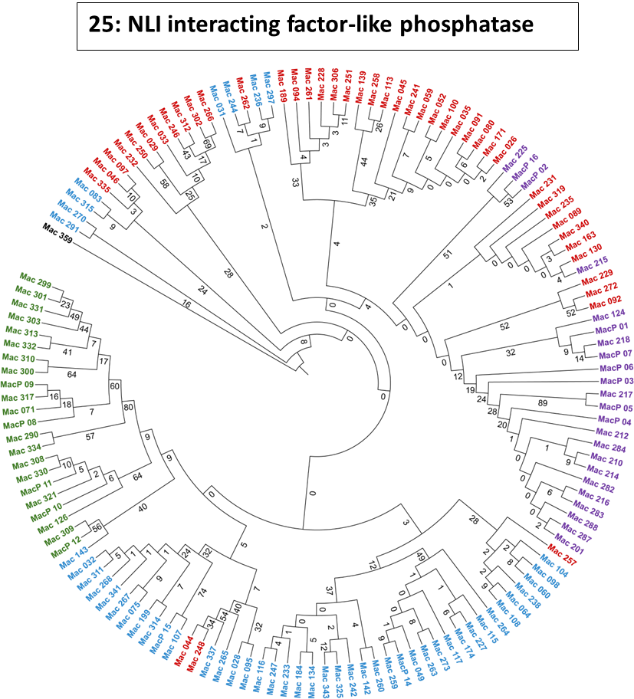

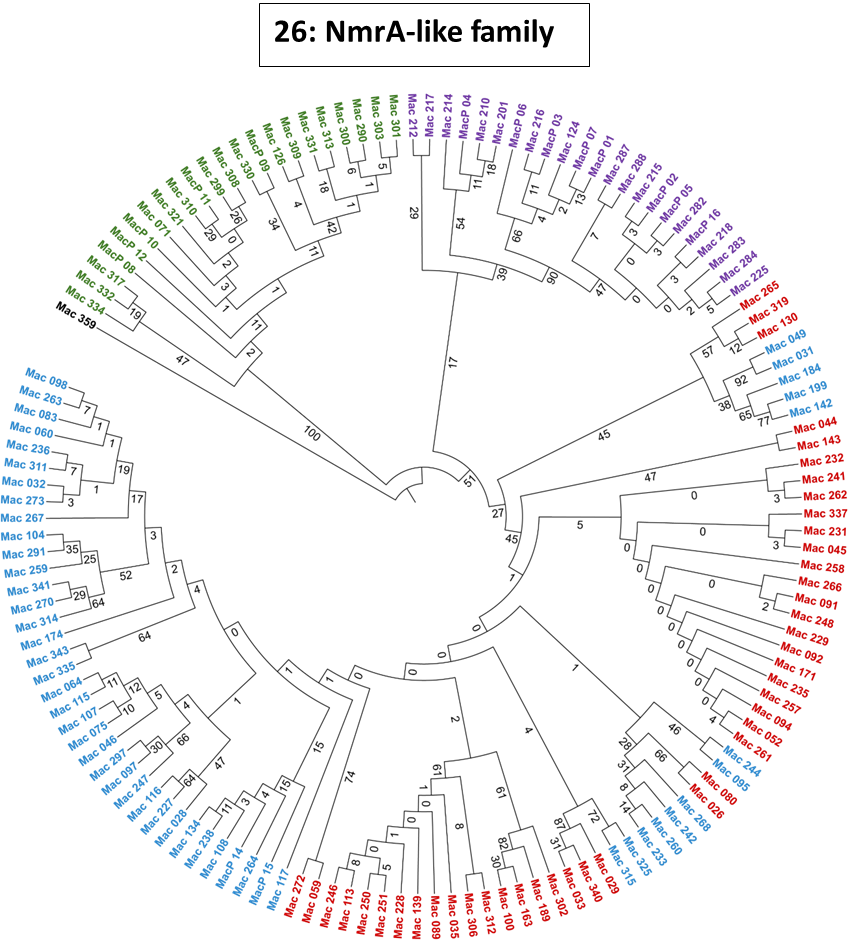

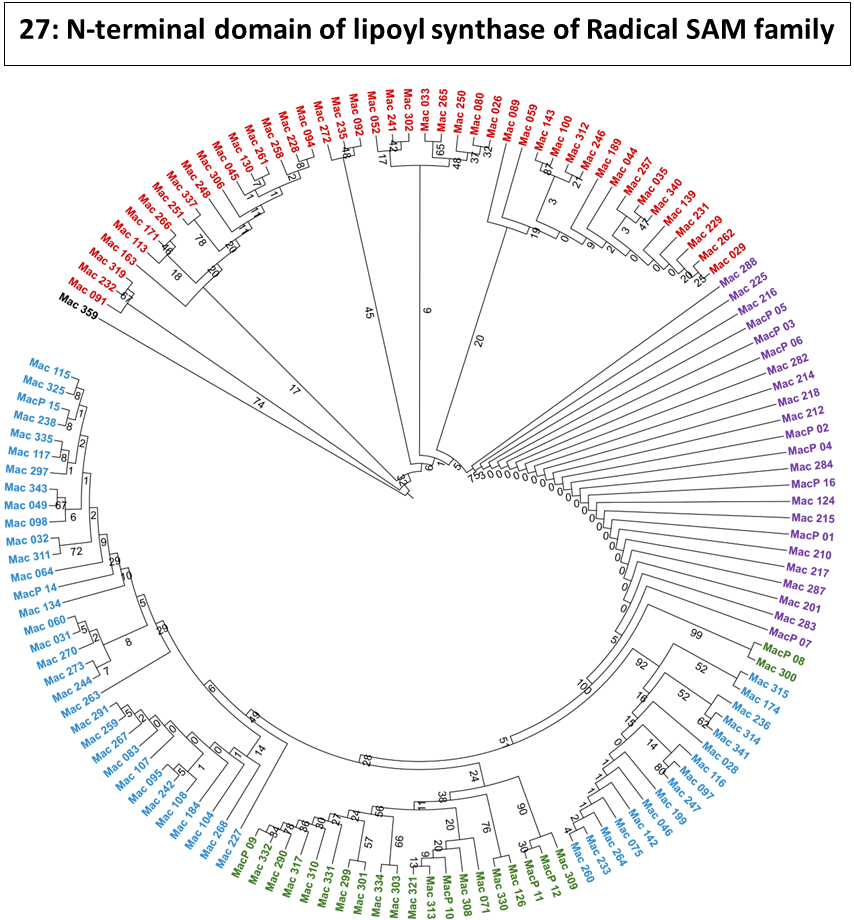

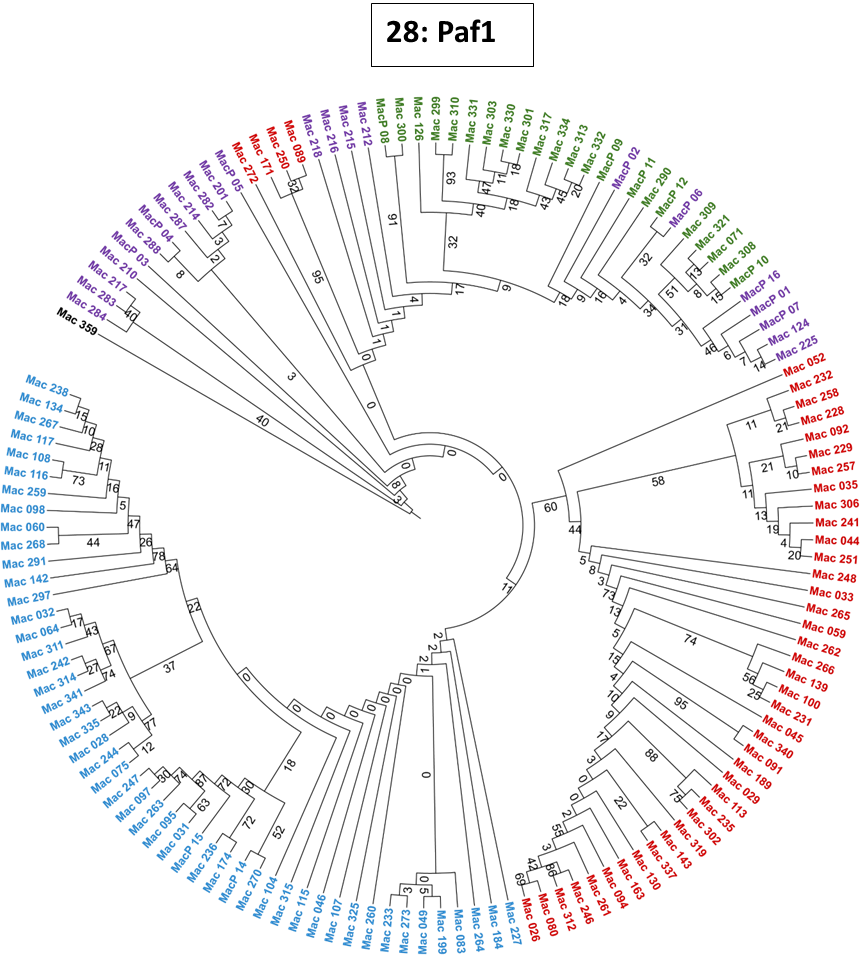


**Figure 9: Individual gene trees constructed for 45 nuclear gene CDS sequences (Gene trees 25-28)**. Light blue: *M. tetraphylla*, Purple: *M. jansenii*, Red: *M. integrifolia,* Green: *M. ternifolia and Black: L. whelanii*. Numbers above the lines represent ML bootstrap support. Phylogenetic tree constructed using 1000 bootstrap replicates. Accessions were colour coded according to the species.


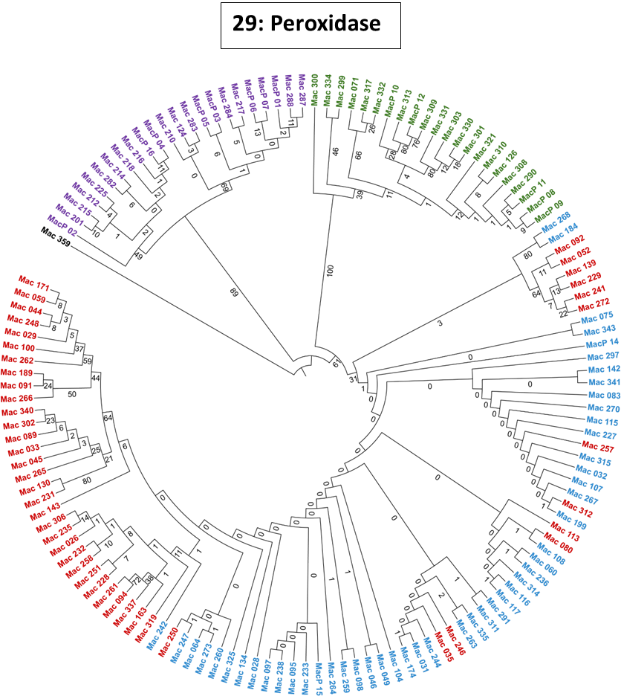

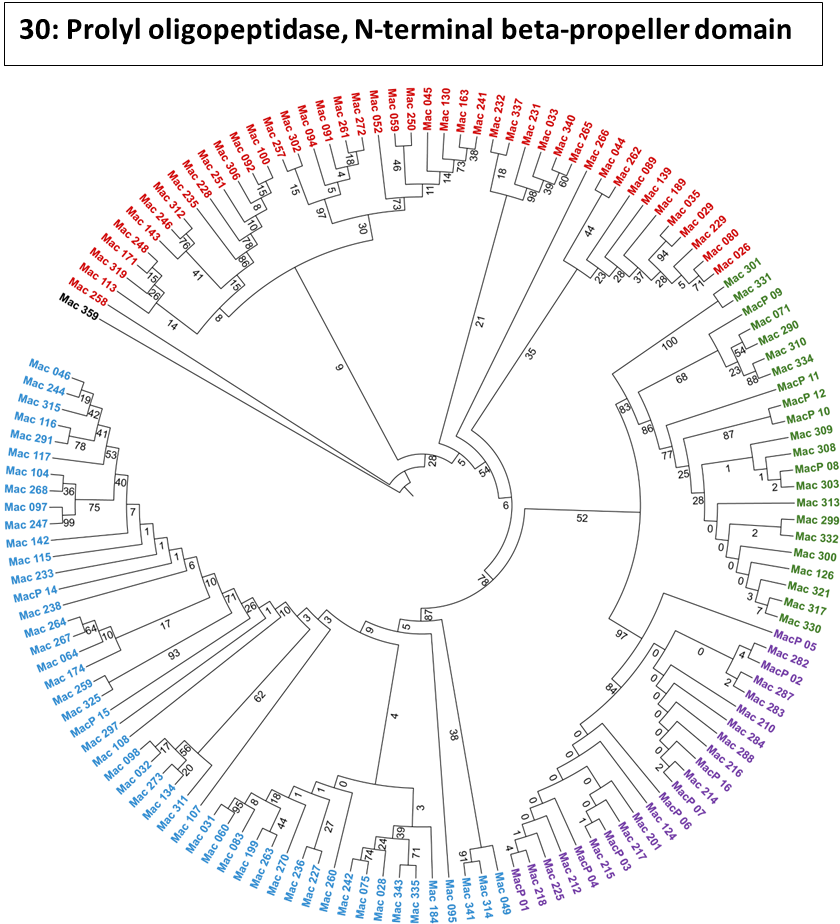

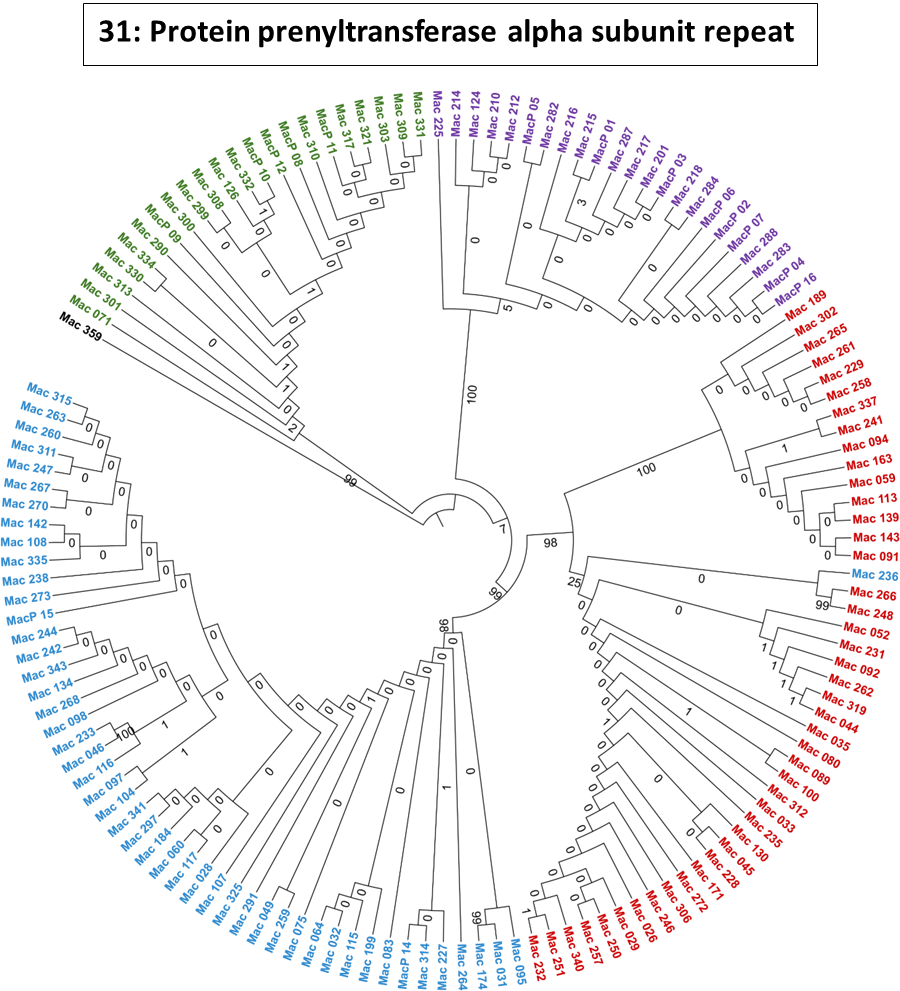

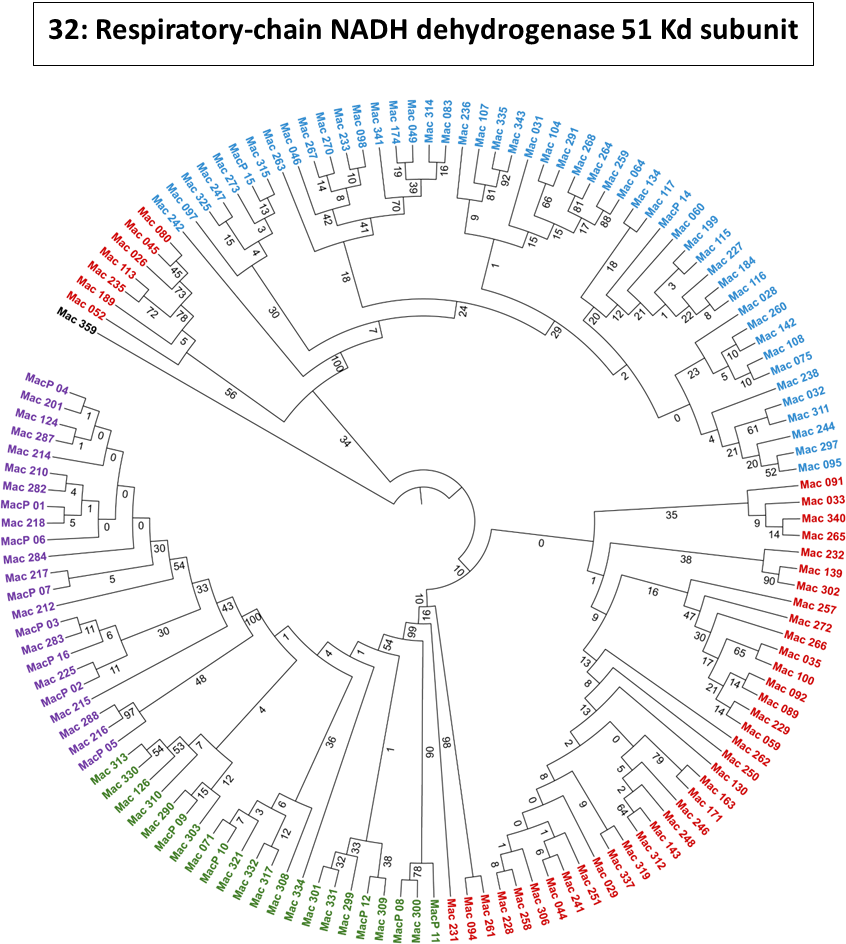


**Figure 9: Individual gene trees constructed for 45 nuclear gene CDS sequences (Gene trees 29-32)**. Light blue: *M. tetraphylla*, Purple: *M. jansenii*, Red: *M. integrifolia,* Green: *M. ternifolia and Black: L. whelanii*. Numbers above the lines represent ML bootstrap support. Phylogenetic tree constructed using 1000 bootstrap replicates. Accessions were colour coded according to the species.


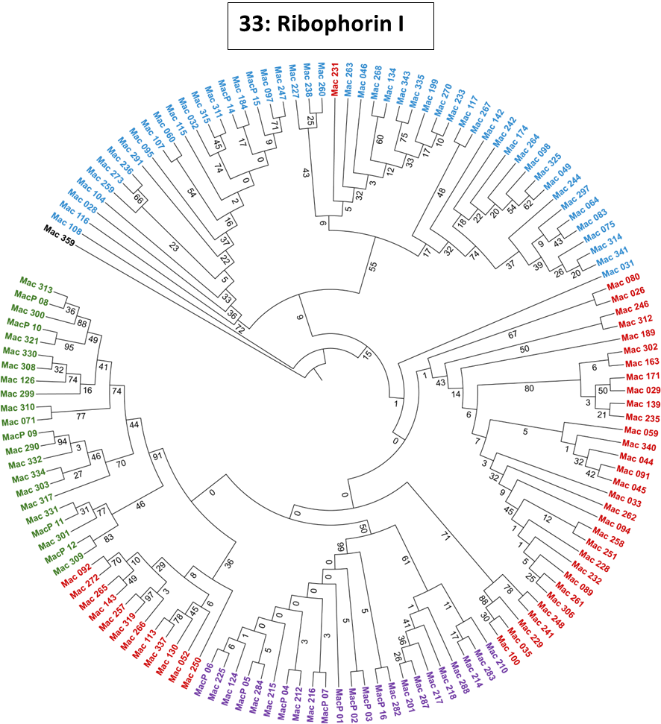

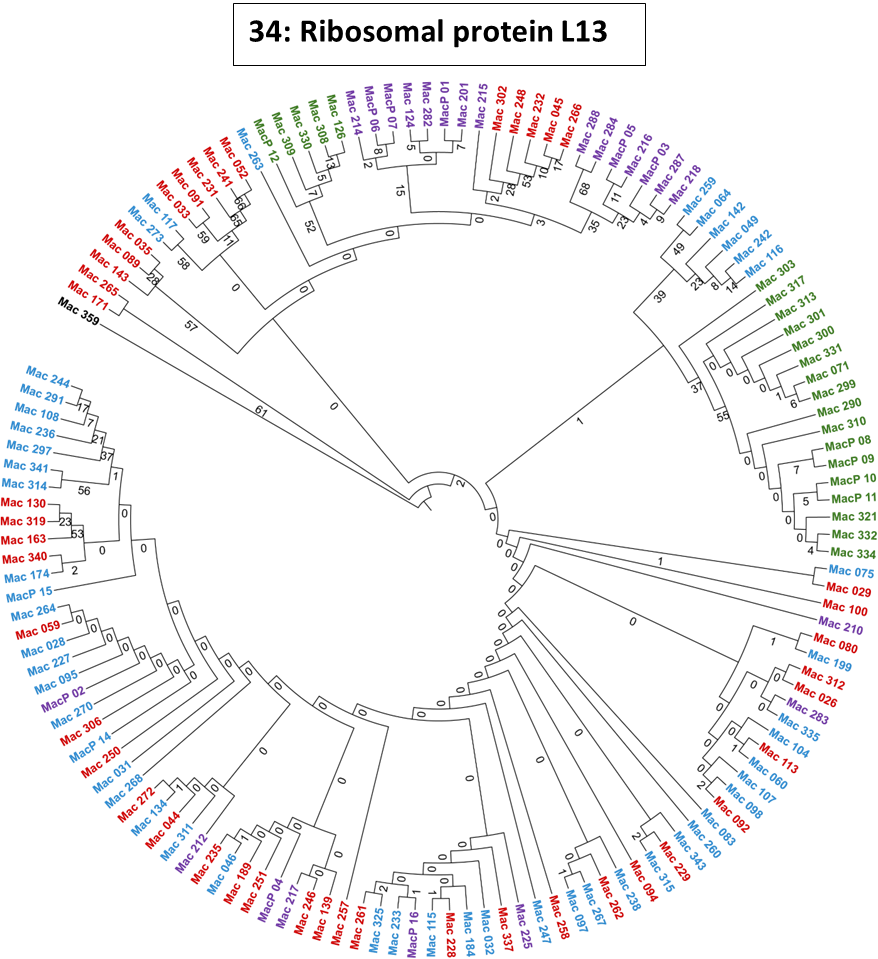

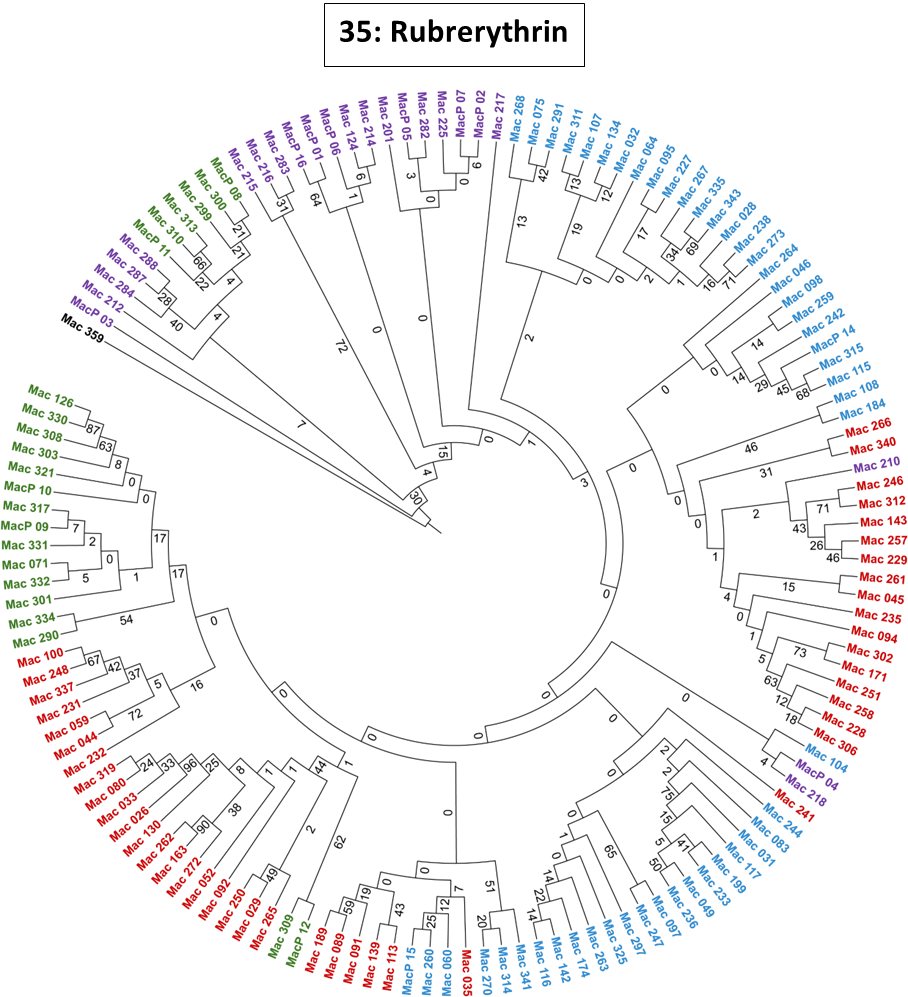

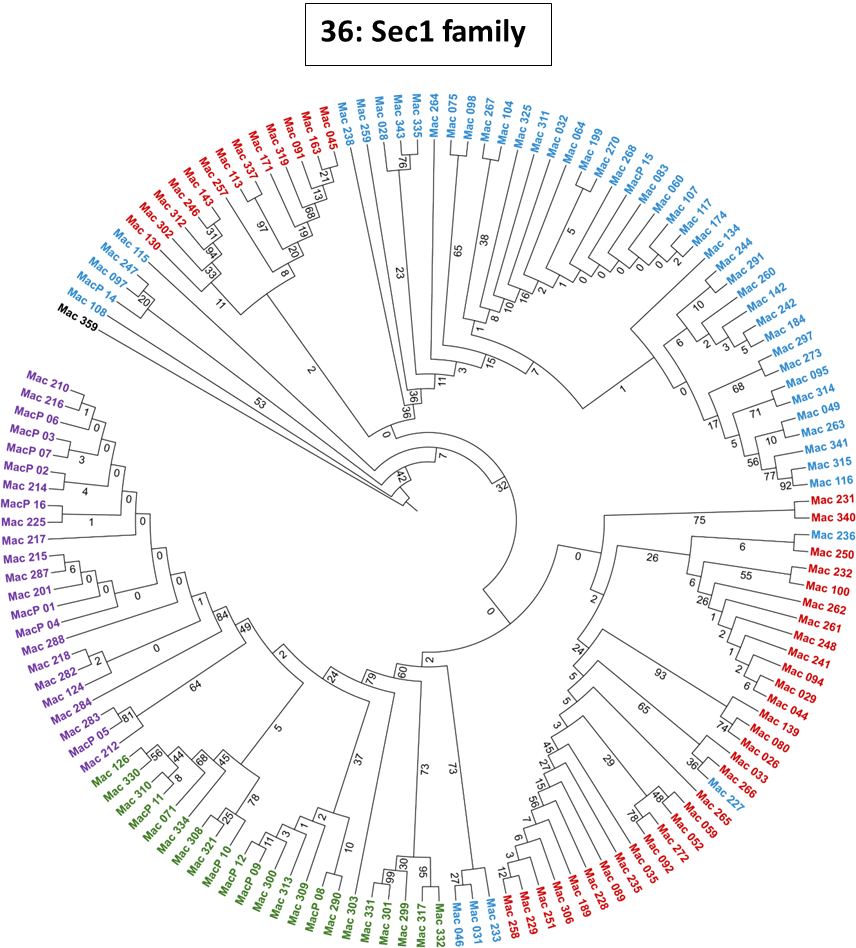


**Figure 9: Individual gene trees constructed for 45 nuclear gene CDS sequences (Gene trees 33-36)**. Light blue: *M. tetraphylla*, Purple: *M. jansenii*, Red: *M. integrifolia,* Green: *M. ternifolia and Black: L. whelanii*. Numbers above the lines represent ML bootstrap support. Phylogenetic tree constructed using 1000 bootstrap replicates. Accessions were colour coded according to the species.


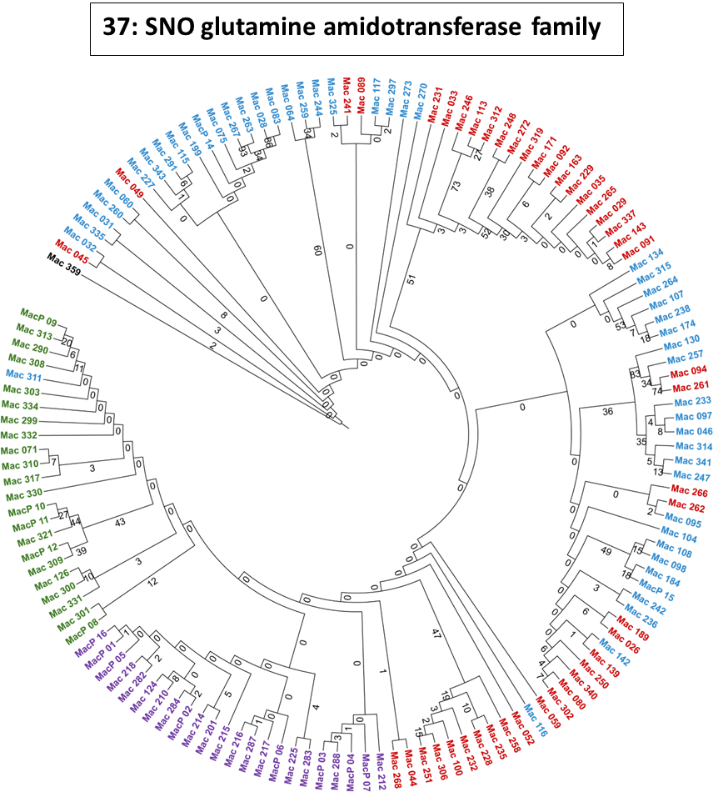

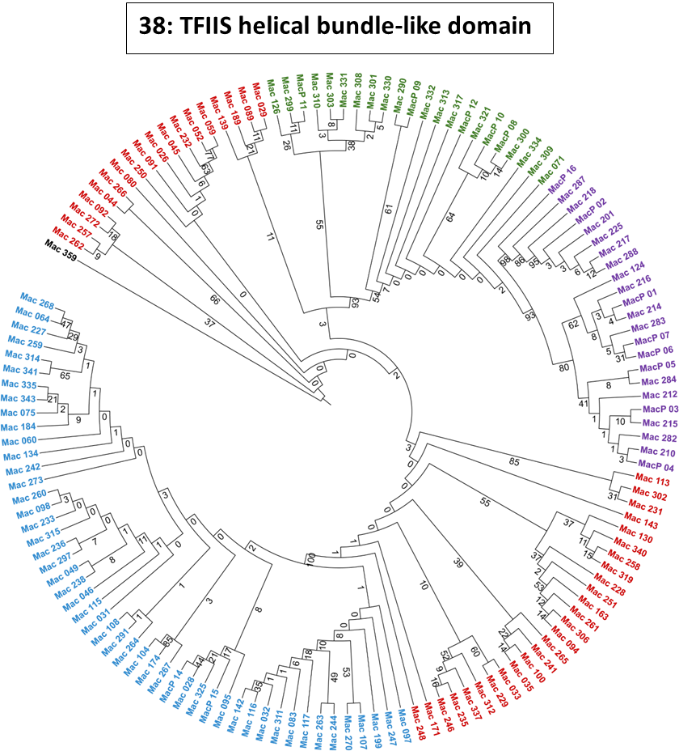

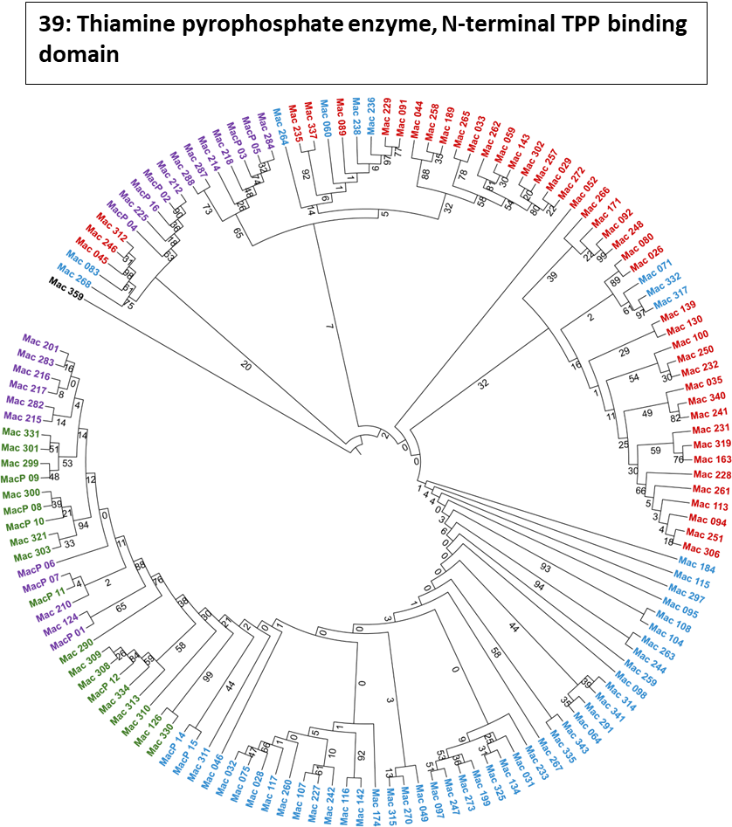

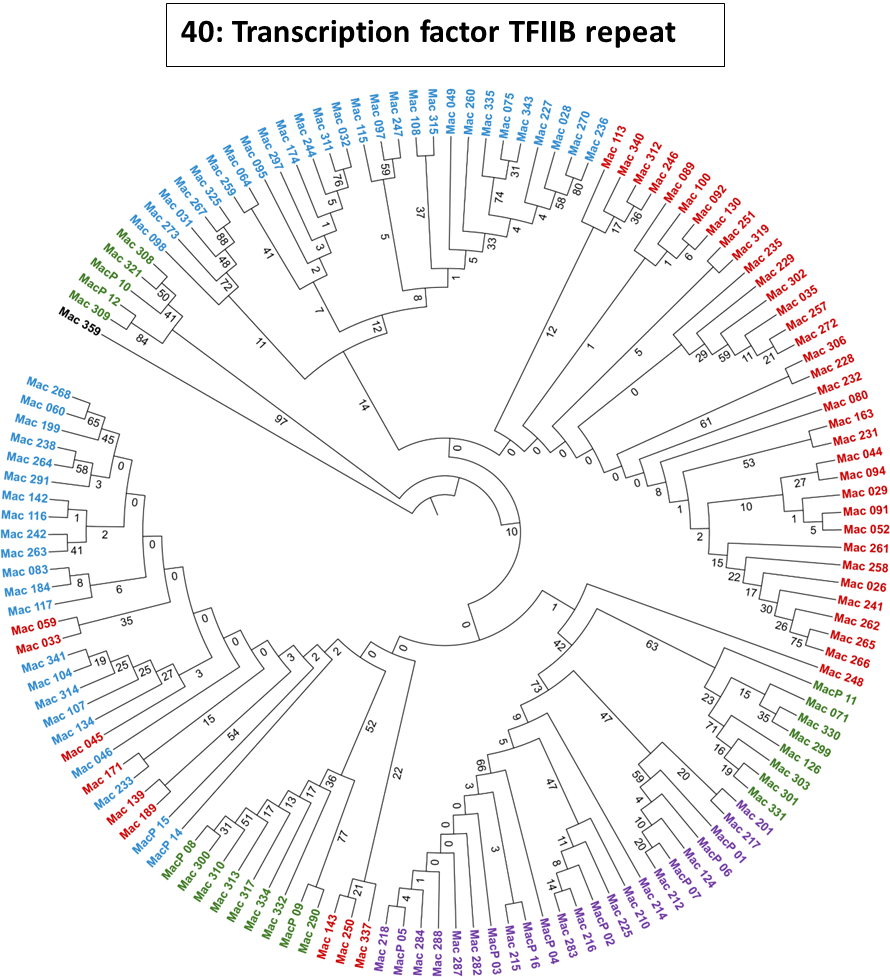


**Figure 9: Individual gene trees constructed for 45 nuclear gene CDS sequences (Gene trees 37-40)**. Light blue: *M. tetraphylla*, Purple: *M. jansenii*, Red: *M. integrifolia,* Green: *M. ternifolia and Black: L. whelanii*. Numbers above the lines represent ML bootstrap support. Phylogenetic tree constructed using 1000 bootstrap replicates. Accessions were colour coded according to the species.


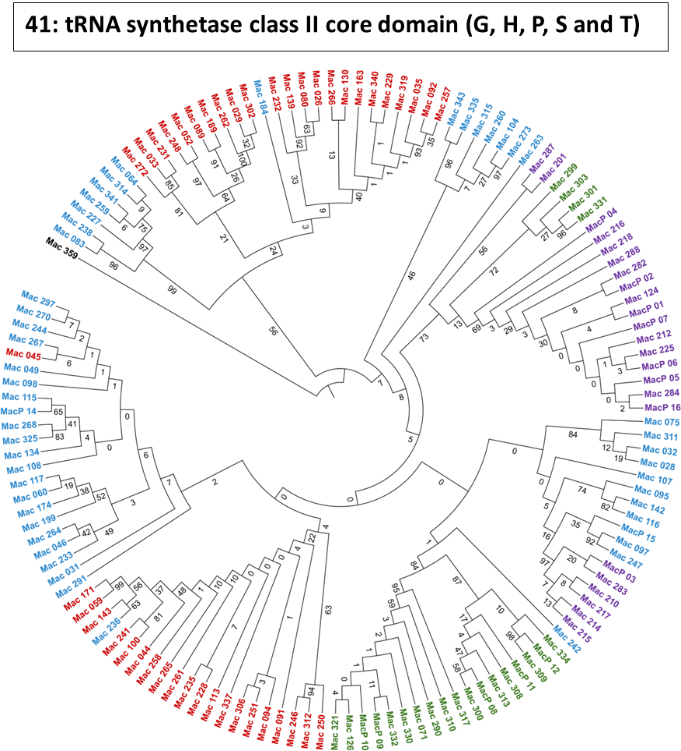

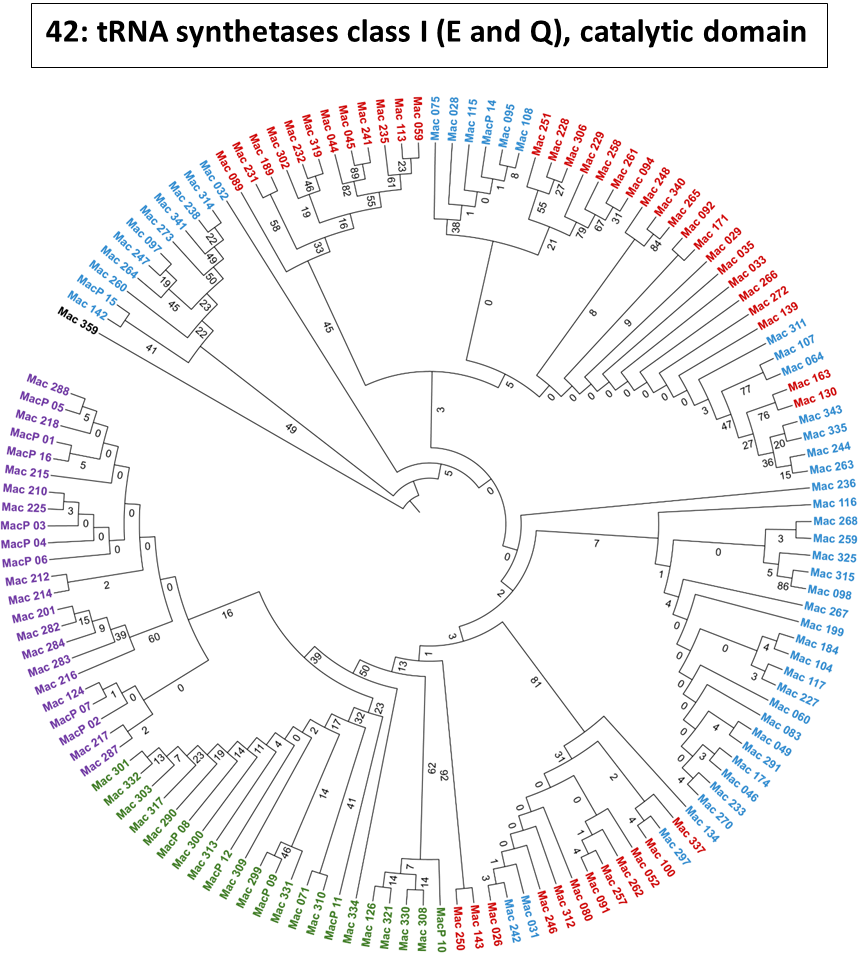

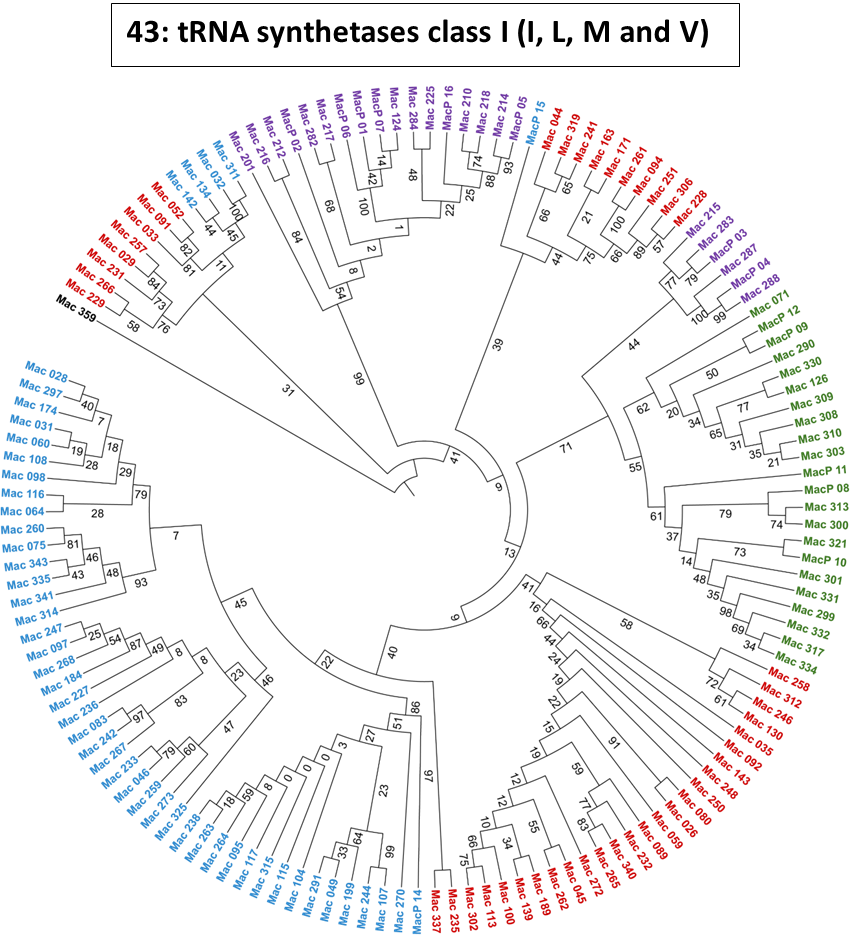

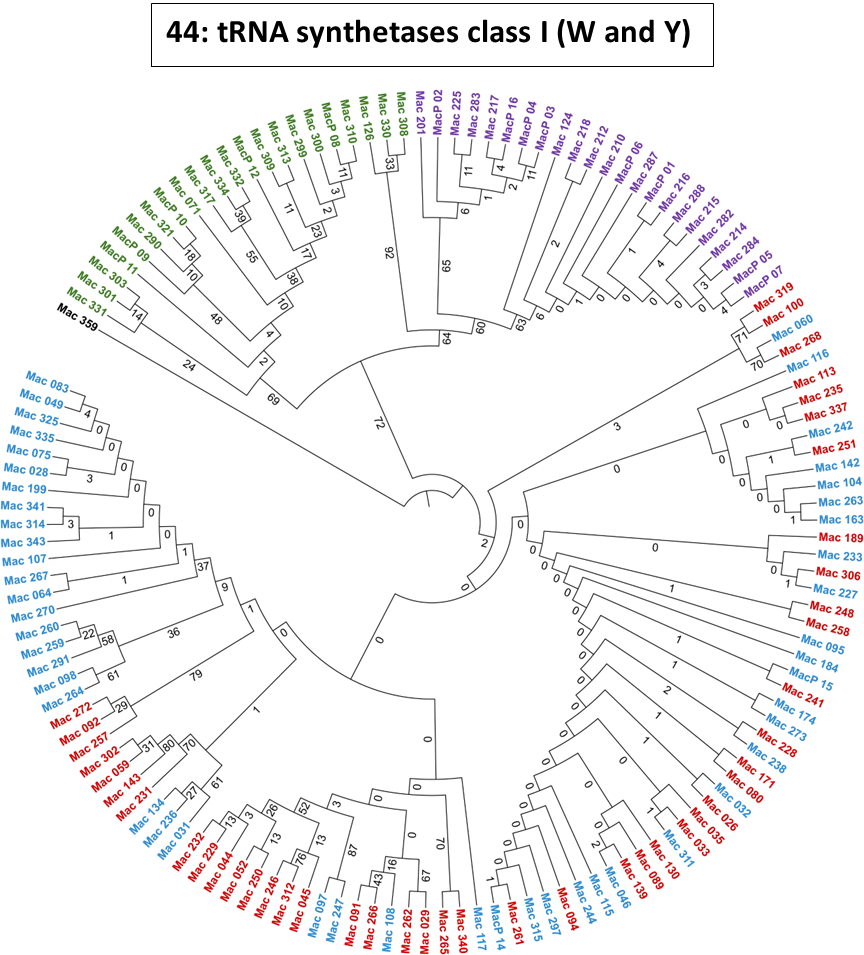


**Figure 9: Individual gene trees constructed for 45 nuclear gene CDS sequences (Gene trees 41-44)**. Light blue: *M. tetraphylla*, Purple: *M. jansenii*, Red: *M. integrifolia,* Green: *M. ternifolia and Black: L. whelanii*. Numbers above the lines represent ML bootstrap support. Phylogenetic tree constructed using 1000 bootstrap replicates. Accessions were colour coded according to the species.


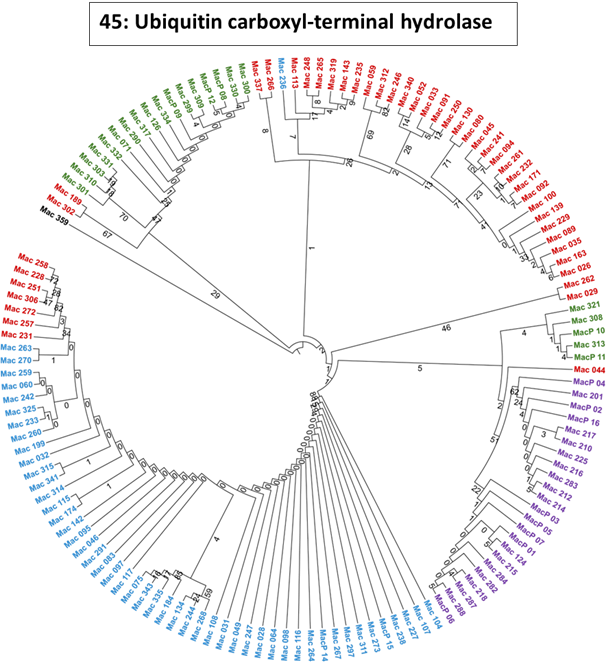


**Figure 9: Individual gene trees constructed for 45 nuclear gene CDS sequences (Gene tree 45)**. Light blue: *M. tetraphylla*, Purple: *M. jansenii*, Red: *M. integrifolia,* Green: *M. ternifolia and Black: L. whelanii*. Numbers above the lines represent ML bootstrap support. Phylogenetic tree constructed using 1000 bootstrap replicates. Accessions were colour coded according to the species.
