# Supplementary material for: Analysis of phylogenetic relationships in Macadamia shows evidence of extensive reticulate evolution
Source: Front Plant Sci. 2024 Oct 15;15:1394244. doi: 10.3389/fpls.2024.1394244 (PMC11518779; doi:10.3389/fpls.2024.1394244)
Supplement: Supplementary file 3 [file DataSheet3.docx]

**Supplemental Information for:**

**Analysis of phylogenetic relationships in *Macadamia* shows evidence of extensive reticulate evolution**

Sachini Lakmini Manatunga^1,2^, Agnelo Furtado^1^, Bruce Topp^3^, Mobashwer Alam^3^, Patrick J. Mason^1,2^, Ardashir Kharabian-Masouleh^1,2^, Robert J Henry^1,2^*

1Queensland Alliance for Agriculture & Food Innovation (QAAFI), University of Queensland, St Lucia QLD 4072, Australia, The University of Queensland, Carmody Rd, St Lucia QLD 4072

2ARC Centre of Excellence for Plant Success in Nature and Agriculture, The University of Queensland, Carmody Rd, St Lucia QLD 4072

3Queensland Alliance for Agriculture & Food Innovation (QAAFI), The University of Queensland, Maroochy Research Facility, Nambour QLD 4560

*Corresponding authors: robert.henry@uq.edu.au


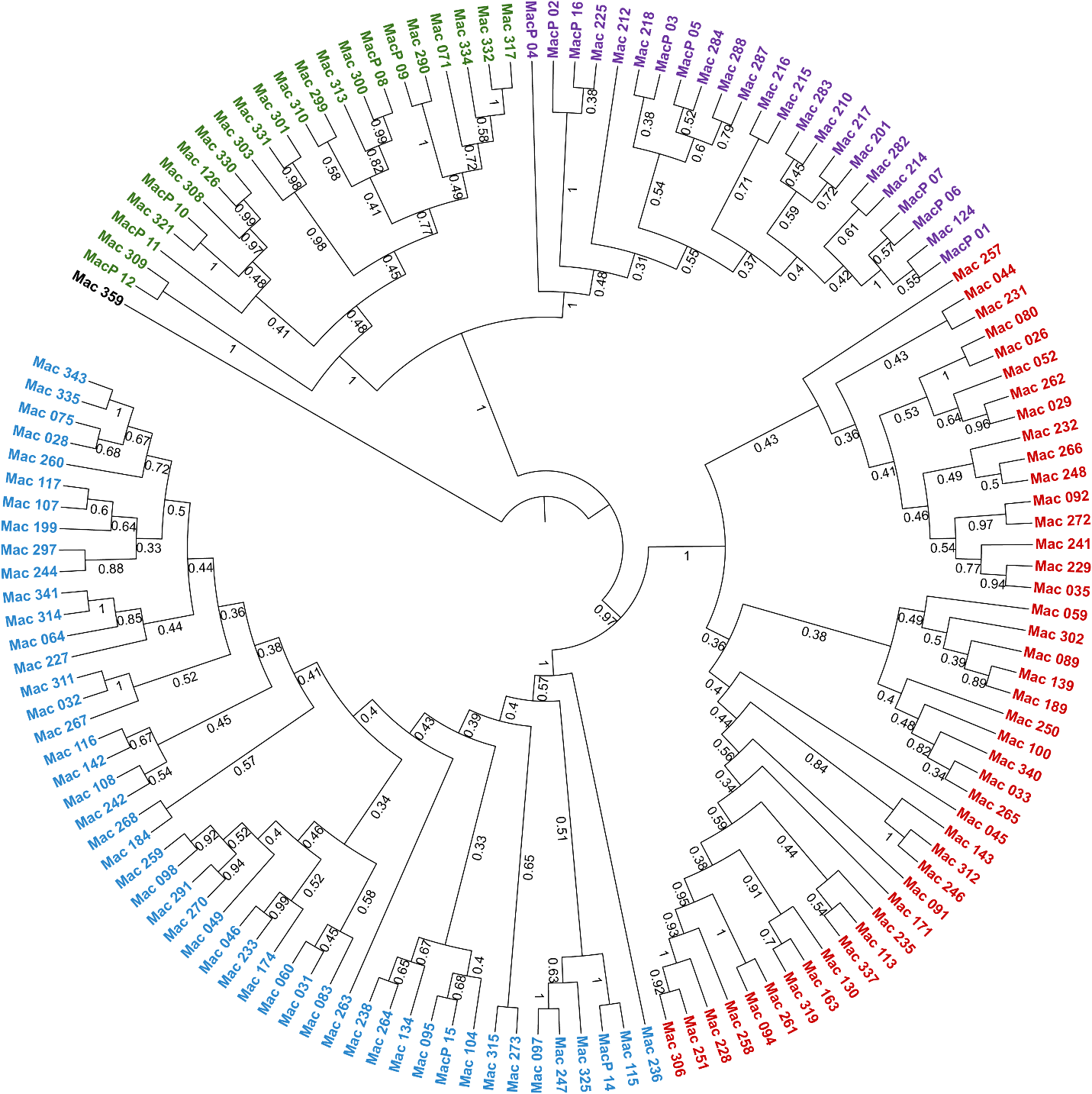


**Figure 10: Single Nucleotide Polymorphisms (SNPs) based phylogeny – (ASTRAL tree)**. Light blue: *M. tetraphylla*, Purple: *M. jansenii*, Red: *M. integrifolia,* Green: *M. ternifolia and Black: L. whelanii*. Numbers associated with branches are local posterior probability values (/1). Accessions were colour coded according to the species.
